# Use of Clinical Pathway Simulation and Machine Learning to Identify Key Levers for Maximizing the Benefit of Intravenous Thrombolysis in Acute Stroke

**DOI:** 10.1161/STROKEAHA.121.038454

**Published:** 2022-07-15

**Authors:** Michael Allen, Charlotte James, Julia Frost, Kristin Liabo, Kerry Pearn, Thomas Monks, Richard Everson, Ken Stein, Martin James

**Affiliations:** Medical School, University of Exeter, St Luke’s Campus, United Kingdom (M.A., C.J., J.F., K.L., K.P., T.M., K.S.).; Computer Science, University of Exeter, Streatham Campus, United Kingdom (R.E.).; Royal Devon and Exeter Hospital, Royal Devon and Exeter NHS Foundation Trust, United Kingdom (M.J.).

**Keywords:** clinical pathways, decision making, hospitals, machine learning, qualitative research

## Abstract

Expert opinion is that about 20% of emergency stroke patients should receive thrombolysis. Currently, 11% to 12% of patients in England and Wales receive thrombolysis, ranging from 2% to 24% between hospitals. The aim of this study was to assess how much variation is due to differences in local patient populations, and how much is due to differences in clinical decision-making and stroke pathway performance, while estimating a realistic target thrombolysis use.

**Methods:**

Anonymised data for 246 676 emergency stroke admissions to 132 acute hospitals in England and Wales between 2016 and 2018 was obtained from the Sentinel Stroke National Audit Programme data. We used machine learning to learn decisions on who to give thrombolysis to at each hospital. We used clinical pathway simulation to model effects of changing pathway performance. Qualitative research was used to assess clinician attitudes to these methods. Three changes were modeled: (1) arrival-to-treatment in 30 minutes, (2) proportion of patients with determined stroke onset times set to at least the national upper quartile, (3) thrombolysis decisions made based on majority vote of a benchmark set of hospitals.

**Results:**

Of the modeled changes, any single change was predicted to increase national thrombolysis use from 11.6% to between 12.3% to 14.5% (clinical decision-making having the most effect). Combined, these changes would be expected to increase thrombolysis to 18.3%, but there would still be significant variation between hospitals depending on local patient population. Clinicians engaged well with the modeling, but those from hospitals with lower thrombolysis use were most cautious about the methods.

**Conclusions:**

Machine learning and clinical pathway simulation may be applied at scale to national stroke audit data, allowing extended use and analysis of audit data. Stroke thrombolysis rates of at least 18% look achievable in England and Wales, but each hospital should have its own target.

**See related article, p 2768**

For ischemic strokes, thrombolysis is an effective treatment for the management of acute stroke if given soon after stroke onset^1^ and is recommended for use in many parts of the world including the United States and Europe. In the 2019 to 2020 stroke national audit of England and Wales,^2^ thrombolysis use was 11.7% overall, with use by individual hospitals ranging from 4.3 to 28.1%. Similar overall rates are observed in the United States. Between 2012 and 2018, thrombolysis use in the United States increased from 6.3% to 11.8%,^3^ and rates of about 11.5% have been maintained since, including through to June 2020 during the COVID pandemic.^4^

The use of targets for thrombolysis varies across the world. The European Stroke Organisation has prepared a European Stroke Action Plan^5^ and has suggested a European target of at least 15% thrombolysis, with median onset-to-needle (also known as onset-to-treatment) times of <120 minutes, noting that evidence suggests that achieving these targets may be aided by centralization of stroke services.^6,7^ In the United Kingdom, the National Health Service (NHS) long-term plan includes a target of 20% of emergency stroke admissions being treated with thrombolysis,^8^ and this is reflected by the NHS Sentinel Stroke National Audit Programme (SSNAP) which monitors stroke care in the United Kingdom (excluding Scotland).^9^ There are no overall targets for thrombolysis use in the United States but the Get With The Guidelines–Stroke program^10^ from the American Heart Association provides a quality improvement tool for hospitals which includes monitoring tools for thrombolysis use and targets such as door-to-needle in 60 minutes. Although there are no specific targets for thrombolysis use in the United States, there have been considerable efforts to improve use and speed of thrombolysis, such as combining expert telemedicine consultation with the use of the Helsinki model for streaming the thrombolysis pathway.^11^

An analysis of the IST-3 trial (The Third International Stroke Trial) for thrombolysis concluded that 60% of ischemic stroke patients arriving within 4 hours of known stroke onset were suitable for thrombolysis.^12^ Assuming 40% of patients arrive within 4 hours of known stroke onset, and assuming 85% of stroke is ischemic, this gives a potential target of 20% thrombolysis (in 2016–2018 in England and Wales, 37% of emergency stroke patients arrived within 4 hours of known stroke onset; see results section of this report).

There is, therefore, still a gap between clinical expert opinion and analysis on target use of thrombolysis, and actual use of thrombolysis.

In work described here, we use machine learning and clinical pathway simulation to ask a series of “what if?” questions about the thrombolysis pathway at each hospital—examining the effect of changing pathway speed or adopting the clinical decision-making of other hospitals. By examining these scenarios, we can produce a realistic target thrombolysis use, and resulting clinical benefit, for each hospital based on the hospital’s own emergency stroke admissions population. We also used qualitative research to understand the potential influence these modeling outputs can have on clinicians, with the aim to best support the maximal appropriate use of thrombolysis and reduce unnecessary variation. Although this work focuses on England and Wales, the methodology should be applicable to other geographies.

## Methods

Original data for this project cannot be shared, but all analysis code and results may be found in an accompanying online book https://samuel-book.github.io/samuel-1 (doi: 10.5281/zenodo.5078131).

The Supplemental Material contains more detail on data access (section 1), data fields (section 2), machine learning (section 3), and clinical pathway simulation (section 4). We followed the Turing Way^13^ for this work‚ and have made available all of the detailed methodology‚ code‚ and results (see the accompanying online book). The Supplemental Material and accompanying online book adhere to the STRESS guidelines (Strengthening the Reporting of Empirical Simulation Studies) for reporting simulation studies^14^ and reporting of the machine learning models followed the TRIPOD guidelines (Transparent Reporting of a Multivariable Prediction Model for Individual Prognosis or Diagnosis).^15^

### Data

Data were obtained from the SSNAP‚ managed through the Healthcare Quality Improvement Partnership. SSNAP has near-complete coverage of all acute stroke admissions in the United Kingdom (outside Scotland). All hospitals admitting acute stroke participate in the audit, and year-on-year comparison with Hospital Episode Statistics confirms estimated case ascertainment of 95% of coded cases of acute stroke. The NHS Health Research Authority decision tool was used to confirm that ethical approval was not required to access the data. Data access was authorized by the UK Healthcare Quality Improvement Partnership (reference HQIP303).

Data were retrieved for 246 676 emergency stroke admissions to acute hospitals in England and Wales between 2016 and 2018 (3 full years). The 62 features retrieved for each patient are given in the Supplemental Material.

### Analysis Environment

All analysis code was written in Python 3.8. Data manipulation, simulation, and general mathematical modeling were done using NumPy^16^ v1.19 and Pandas^17^ v1.2.0. Machine learning libraries used were Tensorflow^18^ v2.2.6, Scikit-Learn^19^ v0.23.2, Seriate v.1.1.2. All charts were produced with MatPlotLib^20^ v3.3.2. All analyses were conducted in Jupyter-Lab^21^ v 2.2.6.

### Machine Learning

Machine learning models were trained to predict whether a patient would receive thrombolysis or not at each hospital. Patients for machine learning prediction were restricted to those arriving within 4 hours of known stroke onset. Machine learning models were built either for individual hospitals or were built to model all hospitals simultaneously with hospitals as a feature. Accuracy was measured using stratified k-fold cross-validation. Two main accuracy measures are reported: % accuracy (the percentage of predictions that were correct) and receiver operator characteristic area under the curve. We also report the highest value of sensitivity and specificity that may be achieved together (the point where sensitivity and specificity curves cross each other as classification threshold is adjusted).

A total of 51 features for pseudonymized patients were extracted from SSNAP for the machine learning. These covered a pseudonymized hospital ID‚ patient characteristics (eg, age band, gender ethnicity); pathway information (eg, onset to arrival minutes, onset known or unknown, mode of arrival, door-to-needle time); patient comorbidities (eg, hypertension, prestroke diabetes, anticoagulant history); National Institutes of Health Stroke Scale; other clinical features (eg, stroke type, transient ischemic attack in the last month); if thrombolysis was given; and reasons for not giving thrombolysis (eg, age, comorbidity, time, onset time unknown). No feature directly informing whether thrombolysis was given (such as reasons for not giving thrombolysis) was used. The full list of features and descriptions can be found in the Supplemental Material.

We report on logistic regression, random forest, and neural network models, as described in more detail in the Supplemental Material. Subsequent work uses hospital-based random forest models. We chose these models due to their good performance, easier explainability to clinicians, and strong hospital independence.

A benchmark set of hospitals was identified by passing the same cohort of 10k patients through all hospital models (this cohort was not used in training of the hospital models). The 30 hospitals with the highest predicted thrombolysis use in this cohort of patients were identified as benchmark hospitals. All hospitals’ patients were passed through this cohort of 30 hospitals to predict thrombolysis use (yes or no) at each benchmark hospital. A majority vote was used to classify a patient as “would receive thrombolysis.”

### Clinical Pathway Simulation

Hyperacute stroke pathways are subject to variation in onset to arrival times, scanning, clinical decision-making, and other factors. We captured this variation using a Monte Carlo simulation model of the clinical pathway. The clinical pathway simulation is based on passing individual virtual patients through a stroke pathway. Each scenario passes ≈8 million patients through the model (100 years of patients through each hospital). To attain the required speed, the pathway simulation was coded using NumPy arrays in Python. Baseline process times (onset to arrival, time to scan, time from scan to treatment) and whether stroke onset time is determined were based on distributions fitted to each hospital’s data. The proportion of patients with known stroke onset was taken from the hospital’s own data. Likelihood to receive thrombolysis if scanned with time to treat in the baseline model was taken from the proportion of patients who were scanned within 4 hours of known stroke onset at each hospital.

**Figure 1.**
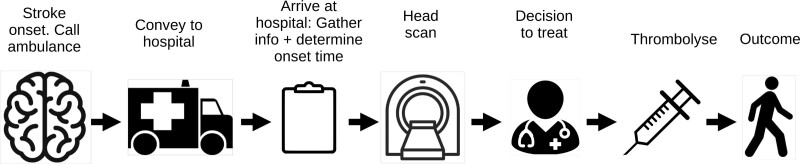
Schematic representation of the stroke pathway as simplified for the simulation.

Key process steps in the pathway are shown in Figure 1. Patients could leave the pathway at each step if their pathway durations exceed the permitted time limits or they become ineligible for treatment. Only patients that satisfied all restrictions continued along the full length of the pathway and received thrombolysis. The outcome was then calculated as a probability of having a good outcome of modified Rankin Scale (mRS) score of 0 to 1. If the patient did not receive thrombolysis the probability of a good outcome was the baseline nonthrombolysed probability of a good outcome in the population age group (aged under 80 years or aged 80+ years). If the patient received thrombolysis‚ then the probability of a good outcome was based on age group and time to treatment using our previously published method.^22^

Three alternative “what if?” scenarios were investigated for each hospital:

1. Base: Uses the hospitals’ recorded pathway statistics in SSNAP.
2. Speed: Sets 95% of patients having a scan within 4 hours of arrival, and all patients have 15 minutes arrival-to-scan time and 15 minutes scan-to-needle time.
3. Onset-known: Sets the proportion of patients with a known stroke onset time to the national upper quartile if currently less than the national upper quartile (leave any greater than the upper national quartile at their current level).
4. Benchmark: The benchmark thrombolysis rate takes the likelihood to give thrombolysis for patients scanned within 4 hours of onset from the majority vote of the 30 hospitals with the highest predicted thrombolysis use in a standard 10k cohort set of patients. These are from hospital-based random forest models.

### Qualitative Research

The overall objective of the qualitative research was to understand influence of modeling, including the use of machine learning techniques, in the context of the national audit, to support efforts to maximize the appropriate use of thrombolysis and reduce unwarranted variation. This was performed by a mixture of face-to-face or remote semi-structured interviews with 19 clinicians (18 medics and one specialist stroke nurse) either in groups or individually. Participants were chosen from hospitals with a range in current thrombolysis use (we sampled equally from tertiles of thrombolysis use: <9.0%, 9.0 to 13.9%, and 14% and above). All data were anonymized. Ethical approval was provided by the UK Health Research Authority and Health and Care Research Wales 19/HRA/5796. A framework analysis of the transcripts was performed with 4 broad objectives:

1. Explore current understanding and rationale for the use of thrombolysis for ischemic stroke, to establish reasons for the variance in the use and speed of thrombolysis.
2. Understand physician perspectives on simulation and machine learning feedback, to influence how simulation can be incorporated into the SSNAP to have a positive impact on practice.
3. Identify potential routes for the implementation of machine learning feedback, to inform and improve future stroke management.
4. Explore how physicians interpret the potential consequences of following changes in pathway suggested by simulation.

(See Supplemental Material section 5 for further methodological details).

## Results

### Machine Learning

The Table shows the accuracy of machine learning models. Models ranged from 81% to 86% accuracy depending on model type. The model with the highest accuracy, by a small margin, was a neural network using embedding layers for hospital ID, clinical features of the patients, and pathway timings. For the remaining experiments‚ we used hospital-level random forest models (with 84.3% accuracy). These models have slightly lower accuracy than other models but have easier explainability and strong hospital independence. Learning curves suggested that the accuracy of the hospital-level random forest models was limited a little by the data size available for each hospital. Although overall accuracy was 84.3%, accuracy reached about 83% with a training set size of at least 500 cases per hospital and dropped below 80% with a training set size of fewer than 125 cases per hospital. Forty-eight percent of hospitals had a training set size of at least 500, and 97% had a training set size of at least 125.

**Table.**
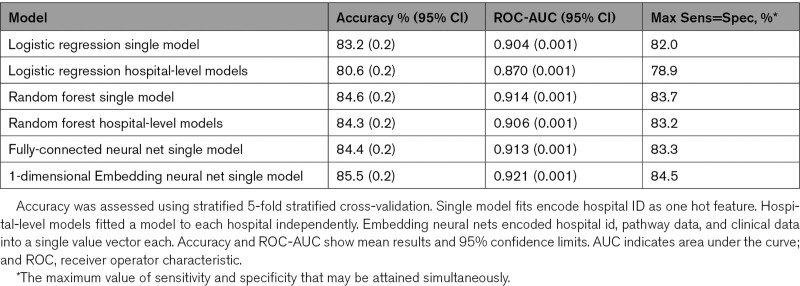
Machine Learning Accuracy

**Figure 2.**
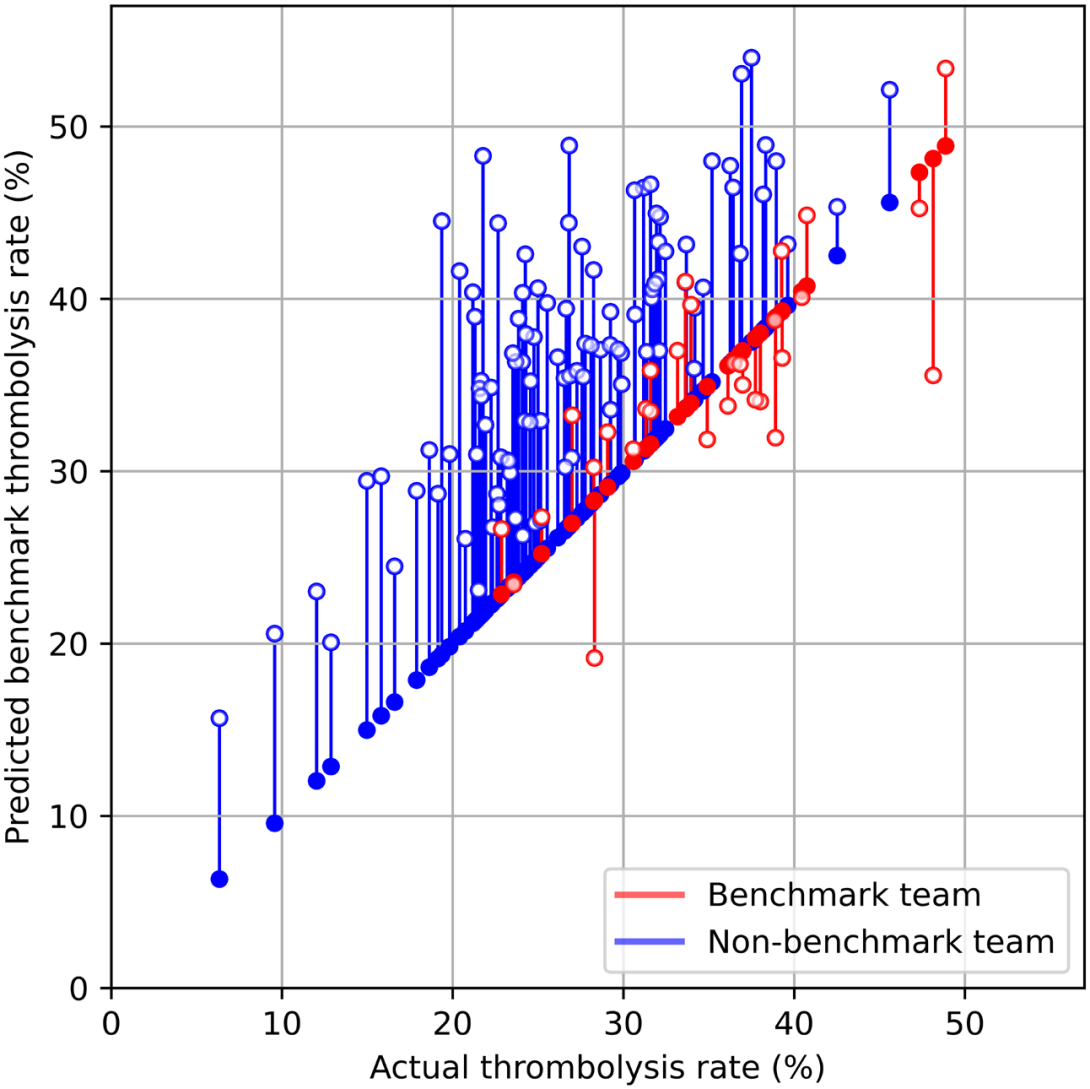
**A comparison of actual thrombolysis rate at each hospital and the predicted thrombolysis rate if decisions were made according to the majority vote of the 30 benchmark hospitals.** Thrombolysis rate is predicted for patients arriving within 4 h of known stroke onset. The solid circle shows the current thrombolysis use, and the open circle shows the thrombolysis use predicted by a majority vote of the benchmark hospitals. The red points are those hospitals that are in the top 30 thrombolysing hospitals (the benchmark set) when cohort thrombolysis use is predicted, with all other hospitals colored blue.

The predicted thrombolysis use at each hospital according to the majority vote of 30 benchmark hospital clinical decision models is shown in Figure 2. When results are weighted by the number of patients attending each hospital, using the benchmark hospital models to decide if a patient would receive thrombolysis, national thrombolysis use would increase from 29.5% to 36.9% of patients arriving within 4 hours of known stroke onset.

When comparing decisions for the 10k cohort predicted by the model, we found that overall, 78% of patients would have a treatment decision agreed by 80% of hospitals. However, there was more agreement around those not to give thrombolysis than those who received thrombolysis: of those who were not given thrombolysis, 85% had agreement by 80% of hospitals, whereas of those who were given thrombolysis, 60% had agreement by 80% hospitals.

### Clinical Pathway Simulation

**Figure 3.**
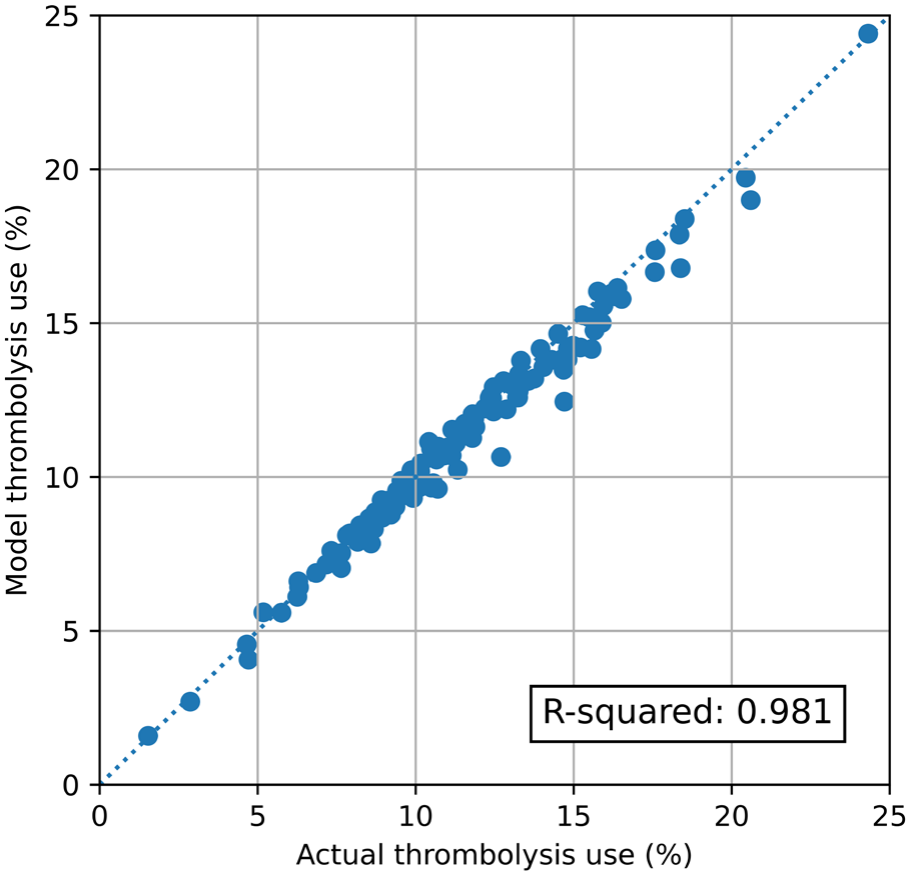
**Validation of the stroke thrombolysis pathway model.** The *x* axis shows the actual thrombolysis use in each hospital (for patients with an out-of-hospital onset stroke), and the *y* axis shows the thrombolysis use predicted from the pathway model. Model parameters were based on pathway statistics for each hospital. The dotted line shows a 1:1 correlation between actual and predicted values.

The pathway model reliably replicated the thrombolysis use in hospitals (Figure 3). Predicted thrombolysis use correlated with actual thrombolysis use with an R-squared of 0.979. The mean thrombolysis use (averaged at hospital level, weighting all hospitals equally) was 11.45% in the observed data, and 11.23% in the pathway model output. The mean difference in thrombolysis use between predicted and actual was 0.22 percentage points. The mean absolute difference in thrombolysis use between predicted and actual was 0.52 percentage points.

**Figure 4.**
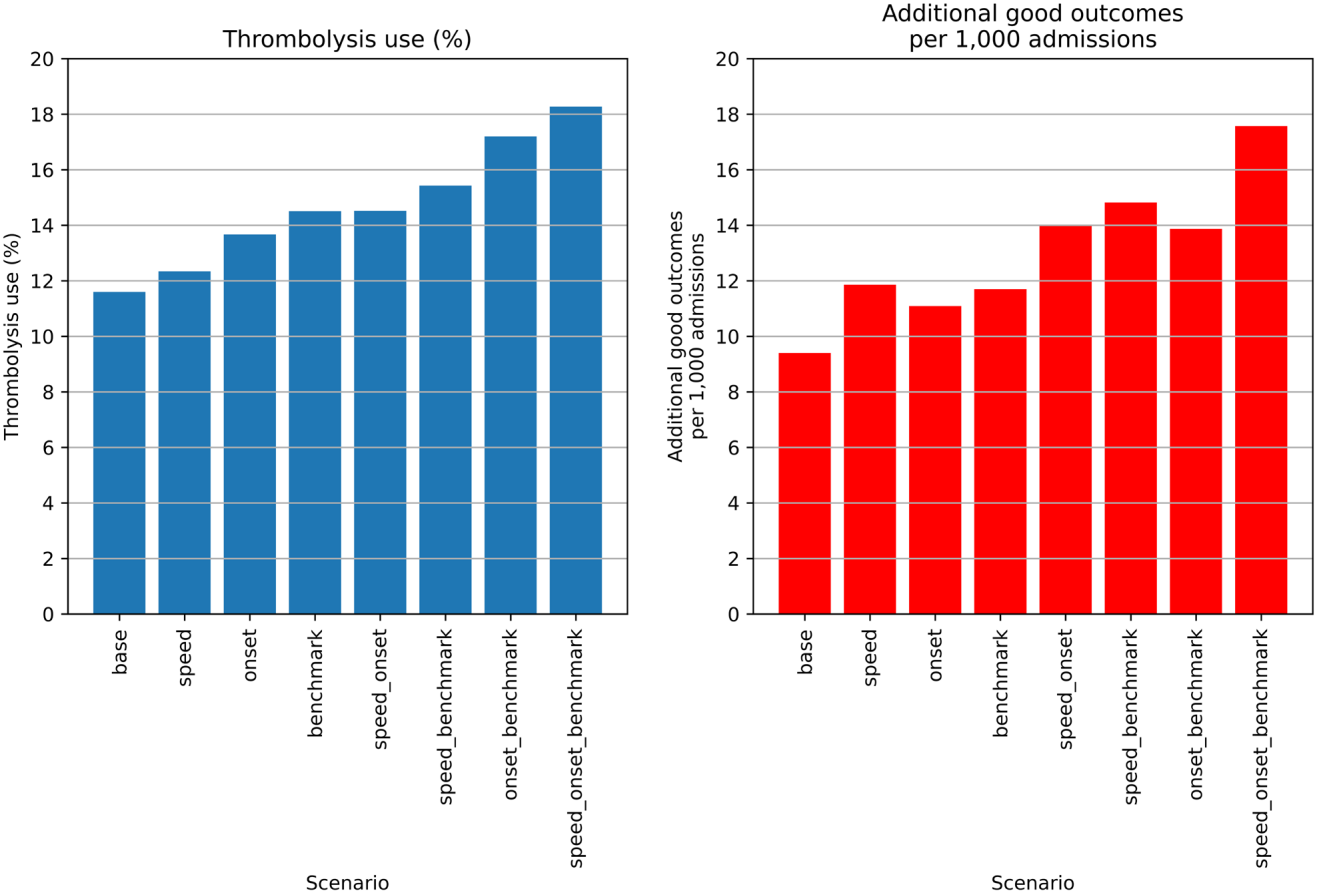
**The effect of changing aspects of the stroke pathway (speed of stroke pathway, determining stroke onset time, and using benchmark decisions) on predicted use of thrombolysis and resulting clinical benefit. Left**, Predicted thrombolysis use (% all of all emergency stroke admissions). **Right**, Clinical benefit (number of additional good outcomes, modified Rankin Scale score of 0–1 at 3–6 mo, per 1000 emergency stroke admissions. Results show effects across all 132 English hospitals, with averages weighted by admission numbers.

Figure 4 shows the overall net effect of separate and combined changes to the stroke pathway. The pathway simulation suggests that thrombolysis use could potentially be increased from 11.6% to 18.3% of all emergency admissions, and the clinical benefit increased from 9.4 to 17.6 additional good outcomes per 1k admissions. The main drivers in improvement in thrombolysis use are benchmark decisions > determining stroke onset > speed, whereas the main drivers in improvement in outcomes are speed > benchmark decisions > determining stroke onset.

**Figure 5.**
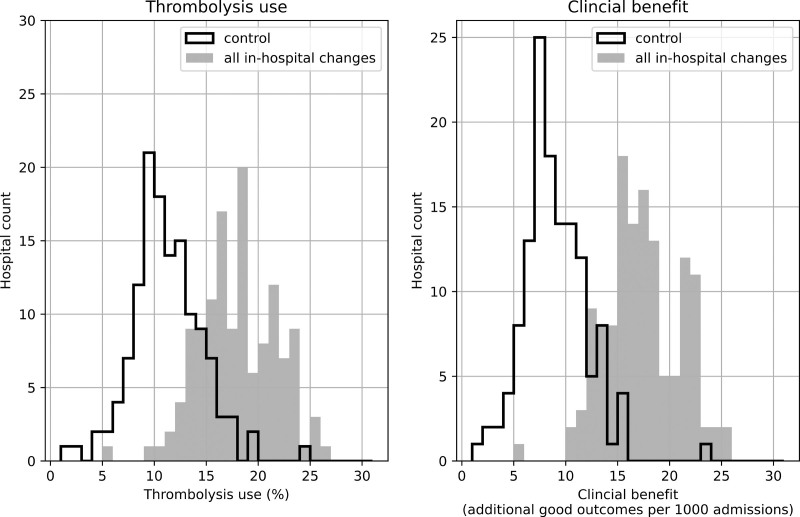
**The effect of combined improvements on predicted thrombolysis use and resulting clinical benefit.** The combined changes were changes to speed (95% of patients have 15 min arrival-to-scan and 15-min scan-to-treatment, with other patients not being scanned within 4 h of arrival), determining stroke onset time (to the national upper quartile if currently lower), and using benchmark decisions. **Left**, Predicted thrombolysis use (% all of all emergency stroke admissions). **Right**, Clinical benefit (number of additional good outcomes, modified Rankin Scale score of 0–1 at 3–6 mo, per 1000 emergency stroke admissions. The unshaded histogram shows the current base-case use of, and benefit from, thrombolysis, and the gray shaded histogram shows the predictions with all 3 changes.

Figure 5 shows the distribution of use of, and benefit from, thrombolysis before and after all the modeled changes. It is noteworthy that there is still significant variation between hospitals but that the distributions have been shifted to the right.

### Qualitative Research

Key findings from the qualitative research were

1. Broadly, those hospitals with higher thrombolysis use engaged more positively with the research, and those with lower thrombolysis use were more cautious.
2. Clinicians from lower thrombolysing hospitals tended to emphasize differences in their patients as the reason for lower thrombolysis. Those in midrate thrombolysis hospitals tended to emphasize access to specialist resources as being key in being able to deliver thrombolysis well. Those in higher thrombolysing hospitals tended to emphasize the work and investment that had gone into establishing a good thrombolysis pathway.
3. Clinicians wanted to see the machine learning models expanded to predict probability of good outcome and adverse effects of thrombolysis.
4. Despite this being a small study, physicians engaged with the machine learning process and outcomes, suggesting ways in which the outputs could be modified for feedback to hospitals and utilized to inform thrombolytic decision-making.

(See Supplemental Material section 5 for detailed qualitative research results).

## Discussion

This work represents a novel approach to understanding the persisting variation that still exists in thrombolysis practice in England and Wales, and to developing new ways of supporting efforts to reduce that variation, and in so doing increase the clinical benefit to people with acute ischemic stroke from thrombolysis. Although our study focuses on England and Wales, the approach should be applicable to other geographies. Not all countries currently collect the level of data that is collected in England and Wales, but we hope that by demonstrating how comprehensive national registry data may be put to use we may help encourage the broader collection of comprehensive clinical registry data sets.

Thrombolysis using alteplase was recommended in the United Kingdom in 2008 for acute ischemic stroke within 4.5 hours of known onset and, initially, usage increased rapidly to over 10% on average nationally.^23^ However since 2013, thrombolysis use has remained static at 11% to 12%.^24^ Furthermore, that national average conceals substantial variation in alteplase use between UK hospitals—as reported here, a 5-fold variation in the overall thrombolysis rate.

Our aim was to use a national stroke clinical registry and state-of-the-art tools of machine learning and clinical pathway simulation to gain a better understanding of what variation is due to processes and decision-making, and what a realistic target of thrombolysis use may be in England and Wales. One of our principal objectives was to develop, through the combination of pathway modeling and machine learning, bespoke outputs for individual hospitals that could be incorporated into routine reporting through national audit, moving away from a single identical target for thrombolysis use for all hospitals. We identified several clear and readily implementable changes to processes and decision-making in hyperacute stroke that together could achieve a 58% increase in the number of patients treated with thrombolysis in the United Kingdom and result in a near-doubling of the clinical benefit from thrombolysis. Full implementation of the pathway changes identified in this study would go a long way towards achieving the stated ambition of the long-term plan for the NHS of bringing the United Kingdom up to among the best in Europe for reperfusion treatment for acute stroke.^8^

Among the changes tested, clinical decision-making had the greatest single effect. Our work, therefore, confirms, and adds to, previous work from discrete choice experiments which showed that clinicians vary in their attitudes, regarding thrombolysis, to individual clinical features of patients.^25^

Despite this, these machine learning techniques could not purport to replace clinical judgment in the decision about treating any individual patient and could not be used to provide a definitive predictive model. The highest performing model, embedding neural networks, achieved 84% sensitivity and specificity simultaneously. The hospital-level random forest model, which had 81% accuracy for the decision to thrombolyse (and could attain 78% sensitivity and specificity simultaneously), identifies agreement among 80% of hospitals in the decision to treat a patient in 60% of cases, and for the decision not to treat, agreement among a similar proportion of hospitals in the decision not to treat in 85% of cases. Apart from anything else, these observations confirm that unanimity in the decision to treat across all 132 hospitals contributing data is highly unusual, although it is easier to find agreement on who not to treat than who to treat. So the outputs from the machine learning can only ever be used probabilistically and to look for general patterns in thrombolysis use that may be used as a stimulus to scrutinize decision-making in audits at a local level. In this respect, it is more useful for the benchmarking process, in which the willingness to thrombolyse in an individual hospital is compared to other hospitals.

Pathway changes have been previously shown to have a significant effect on thrombolysis rates and door-to-needle time, for example, by Meretoja et al^26^ in several different settings.^27^ These projects would indicate that the 30-minute door-to-needle time proposed in our model is not an unrealistic or unachievable target.

A familiar pitfall when addressing clinical variation is that the phrase “if only all sites were as good as the best” is, by definition, an oxymoron and lacks credibility with clinical teams who are far short of the best and/or struggling to improve. We have, therefore, sought to neutralize this pitfall through the use of a much more conservative approach—modeling based either on the typical clinical behavior of just the top 30 hospitals or the top-quartile performance for the acquisition of a known onset time. This presents poorly-performing hospitals with a much more credible and achievable objective—you do not need to be as good as the best, often regarded as unachievable but merely match the performance of a better-than-average site, of which there are many.

Our method also addresses another familiar objection regarding high-performing hospitals that such sites are needlessly thrombolysing mild strokes or even stroke mimics, although the latter are excluded from the SSNAP data used in this study. This issue arose in our linked qualitative work with low thrombolysing hospitals. In our cohort of 10k standardized stroke patients presenting within 4 hours, half of the top 30 thrombolysing hospitals would have a lower thrombolysis rate after benchmarking. The moderating effect of a broad-based machine learning method removes extremes at both ends of the scale but still contributes to a substantial increase in the overall thrombolysis rate for nearly all sites and a correspondingly greater population benefit. It would seem not unreasonable, therefore, to anticipate an achievable national thrombolysis rate for patients presenting within 4 hours to be 36.9% compared to the current figure of 29.5%.

### Limitations

A limitation of the study is the amount of data available per individual. Although the current data are sufficient to predict the use of thrombolysis with 85% accuracy, there are sure to be factors influencing decision-making that are not included in the data set. This limitation does not negate the use of these models to explore patterns of thrombolysis use but underly the importance that these tools should not be used for individual case decision-making.

Our predictions about differences in clinical decision-making are necessarily at hospital level. We pick up on general differences in attitude to thrombolysis between hospitals‚ but we cannot detect differences that exist between individual clinicians (as decision-making is the end result of a process, and maybe collective, it may be that decisions can never be fully assigned to an individual). For this work, we were not able to identify the hospital and so could not look at relationships with factors, such as rurality, or organization factors, such as whether a hospital employs a specialist stroke nurse to facilitate the emergency stroke pathway.

The machine learning model described only predicts use of thrombolysis and does not predict outcomes directly. In future work, we plan to use machine learning also to predict outcomes (such as death, mRS on discharge, and thrombolysis-induced hemorrhage; such quality outcomes will also help assess the risk of treatment worsening outcomes in individual patients). We predict likelihood of a good outcome based on clinical trial meta-analysis on the relationship between time to thrombolysis (if given) and the probability of a good outcome, measured as having an mRS of 0-1 at 3 to 6 months. It should be noted that this reflects an excellent disability-free outcome and does not incorporate all the benefit of thrombolysis (such as if a patient were improved from an mRS of 5 to an mRS of 4).

The remaining limitations relate to the potential for implementation. Our qualitative substudy identified the paradox that it was the confident thrombolysing physicians who were most open to the influence of machine learning and other methods of quality improvement but who also needed it the least. Successfully engaging with a large and disparate group of middling-to-low thrombolysing hospitals and clinicians less open to these methods for improvement presents significant challenges and could blunt the impact and resultant benefits. Further research is likely to help address how the concerns of the lower thrombolysing hospitals may be best addressed.

### Implications for Healthcare

Overall, our results suggest that England and Wales can get close to the target of 20% of emergency stroke admissions receiving thrombolysis, but this should not be seen as a single target for all hospitals. Realistically achievable thrombolysis use depends on local patient populations, so a universal target of 20% across all hospitals may overestimate what is achievable at some hospitals while underestimating what is achievable at other hospitals. Local agreed targets may be more appropriate.

The tools developed here have the potential to add further depth of analysis to the national stroke audit outputs, providing each hospital with more in-depth analysis of what an achievable use of thrombolysis may be in their hospital, and what changes to pathway or decision-making would help drive most improvement.

### Conclusions

Machine learning and clinical pathway simulation may be applied at scale to national audit data, allowing extended use and analysis of audit data. These models may help hospitals identify what would most improve benefit from thrombolysis use (if improvement is needed) and identify realistic targets for hospitals given their own patient populations. We can identify patterns of differences in clinical decision-making between hospitals.

Our models have good accuracy. Decision-making can be predicted with 85% accuracy for those patients with a chance of receiving thrombolysis (arriving within 4 hours of stroke onset). This accuracy enables us to look for patterns in clinical decision-making in and between hospitals. Clinical pathway simulation predicts hospital thrombolysis use with an average absolute error of 0.5 percentage points.

Stroke thrombolysis rates of at least 18% look achievable in England and Wales, but each hospital should have its own target.

## Article Information

### Acknowledgments

The authors thank our public and patient involvement representative, Leon Farmer and Penny Thompson, who were involved throughout the project and who helped the project develop deeper understanding and better ways of communicating results.

### Sources of Funding

This report is independent research funded by the National Institute for Health and Care Research (NIHR) Applied Research Collaboration South West Peninsula and the NIHR Health and Social Care Delivery Research (HSDR) Programme. The views expressed in this publication are those of the author(s) and not necessarily those of the NIHR or the Department of Health and Social Care.

### Disclosures

None.

### Supplemental Material

Supplemental Methods

Tables S1–S5

Figures S1–S11

References 28–36

## Supplementary Material



## References

[1] Emberson J Lees KR Lyden P Blackwell L Albers G Bluhmki E Brott T Cohen G Davis S Donnan G; Stroke Thrombolysis Trialists’ Collaborative Group. Effect of treatment delay, age, and stroke severity on the effects of intravenous thrombolysis with alteplase for acute ischaemic stroke: a meta-analysis of individual patient data from randomised trials. Lancet. 2014;384:1929–1935. doi: 10.1016/S0140-6736(14)60584-5 25106063 10.1016/S0140-6736(14)60584-5 PMC4441266

[2] Sentinel Stroke National Audit. Apr2019Mar2020-AnnualResultsPortfolio. Accessed Jun1 1, 2021. https://www.strokeaudit.org/Documents/National/Clinical/Apr2019Mar2020/Apr2019Mar2020-AnnualResultsPortfolio.aspx.

[3] Anand SK Benjamin WJ Adapa AR Park JV Wilkinson DA Daou BJ Burke JF Pandey AS. Trends in acute ischemic stroke treatments and mortality in the United States from 2012 to 2018. Neurosurg Focus. 2021;51:E2. doi: 10.3171/2021.4.FOCUS21117 10.3171/2021.4.FOCUS21117 34198248

[4] Srivastava PK Zhang S Xian Y Xu H Rutan C Alger HM Walchok JG Williams JH de Lemos JA Decker-Palmer MR. Treatment and outcomes of patients with ischemic stroke during COVID-19: an analysis from get with the guidelines-stroke. Stroke. 2021;52:3225–3232. doi: 10.1161/STROKEAHA.120.034414 34192897 10.1161/STROKEAHA.120.034414 PMC8478095

[5] Norrving B Barrick J Davalos A Dichgans M Cordonnier C Guekht A Kutluk K Mikulik R Wardlaw J Richard E. Action plan for stroke in Europe 2018-2030. Eur Stroke J. 2018;3:309–336. doi: 10.1177/2396987318808719 31236480 10.1177/2396987318808719 PMC6571507

[6] Bray BD Campbell J Cloud GC Hoffman A Tyrrell PJ Wolfe CD Rudd AG; Intercollegiate Stroke Working Party Group. Bigger, faster? Associations between hospital thrombolysis volume and speed of thrombolysis administration in acute ischemic stroke. Stroke. 2013;44:3129–3135. doi: 10.1161/STROKEAHA.113.001981 24052511 10.1161/STROKEAHA.113.001981

[7] Lahr MM Luijckx GJ Vroomen PC van der Zee DJ Buskens E. Proportion of patients treated with thrombolysis in a centralized versus a decentralized acute stroke care setting. Stroke. 2012;43:1336–1340. doi: 10.1161/STROKEAHA.111.641795 22426467 10.1161/STROKEAHA.111.641795

[8] NHS. The NHS Long Term Plan. Accessed Jun1 1, 2021. https://www.longtermplan.nhs.uk/wp-content/uploads/2019/08/nhs-long-term-plan-version-1.2.pdf.

[9] HQIP. Sentinel Stroke National Audit Programme - Annual Report 2019-20. Accessed Jun1 1, 2021. https://www.hqip.org.uk/resource/sentinel-stroke-national-audit-programme-annual-report-2019-20/.

[10] American Heart Association. Get With The Guidelines®– Stroke. Accessed February 2, 2022. https://www.heart.org/en/professional/quality-improvement/get-with-the-guidelines/get-with-the-guidelines-stroke.

[11] Nguyen-Huynh MN Klingman JG Avins AL Rao VA Eaton A Bhopale S Kim AC Morehouse JW Flint AC KPNC Stroke FORCE Team. Novel telestroke program improves thrombolysis for acute stroke across 21 hospitals of an integrated healthcare system. Stroke. 2018;49:133–139. doi: 10.1161/STROKEAHA.117.018413 29247142 10.1161/STROKEAHA.117.018413 PMC5753819

[12] Bembenek J Kobayashi A Sandercock P Czlonkowska A. How many patients might receive thrombolytic therapy in the light of the ECASS-3 and IST-3 data? Int J Stroke. 2010;5:430–431. doi: 10.1111/j.1747-4949.2010.00479.x 20854633 10.1111/j.1747-4949.2010.00479.x

[13] The Turing Way Community. The Turing Way: A handbook for reproducible data science. Accessed Jun1 1, 2021. https://the-turing-way.netlify.app/

[14] Monks T Currie CSM Onggo BS Robinson S Kunc M Taylor SJE. Strengthening the reporting of empirical simulation studies: Introducing the STRESS guidelines. J Simul. 2019;13:55–67. doi: 10.1080/17477778.2018.1442155

[15] Collins GS Reitsma JB Altman DG Moons KGM. Transparent reporting of a multivariable prediction model for individual prognosis or diagnosis (TRIPOD): the TRIPOD statement. BMJ. 2015;350:W1–73. doi: 10.7326/M14-0698 10.1136/bmj.g7594 25569120

[16] Harris CR Millman KJ van der Walt SJ Gommers R Virtanen P Cournapeau D Wieser E Taylor J Berg S Smith NJ. Array programming with NumPy. Nature. 2020;585:357–362. doi: 10.1038/s41586-020-2649-2 32939066 10.1038/s41586-020-2649-2 PMC7759461

[17] McKinney W. pandas: a foundational Python library for data analysis and statistics. Python High Perform Sci Comput. 2011;14:1–9.

[18] Abadi M Agarwal A Barham P Brevdo E Chen Z Citro C Corrado GS Davis A Dean J Devin M. Tensorflow: Large-scale machine learning on heterogeneous distributed systems. arXiv. 2016:1603.04467. doi: 10.48550/arXiv.1603.04467

[19] Pedregosa F Varoquaux G Gramfort A Michel V Thirion B Grisel O Blondel M Prettenhofer P Weiss R Dubourg V. Scikit-learn: machine learning in Python. J Mach Learn Res. 2011;12:2825–2830. doi: 10.5555/1953048.2078195

[20] Hunter JD. Matplotlib: a 2D graphics environment. Comput Sci Eng. 2007;9:90–95. doi: 10.1109/MCSE.2007.55

[21] Kluyver T Ragan-Kelley B Pérez F Granger B Bussonnier M Frederic J Kelley K Hamrick J Grout J Corlay S. Jupyter Notebooks -- a publishing format for reproducible computational workflows. Loizides F Schmidt B, eds. In: Positioning and Power in Academic Publishing: Players, Agents and Agendas. Amsterdam, the Netherlands: IOS Press. 2016: 87–90.

[22] Allen M Pearn K Stein K James M. Estimation of stroke outcomes based on time to thrombolysis and thrombectomy. medRxiv. 2020:2020-07. doi: 10.1101/2020.07.18.20156653

[23] Royal College of Physicians. How Good is Stroke Care? The First SSNAP Annual Report (2014). Accessed Jun1 1, 2021. https://www.strokeaudit.org/Documents/National/Clinical/Apr2013Mar2014/Apr2013Mar2014-AnnualReport.aspx.

[24] Sentinel Stroke National Audit Programme. Moving the Dial of Stroke Care: The 6th SSNAP National Report (2019). Accessed Jun1 1, 2021. https://www.strokeaudit.org/Documents/National/Clinical/Apr2018Mar2019/Apr2018Mar2019-AnnualReport.aspx.

[25] De Brún A Flynn D Ternent L Price CI Rodgers H Ford GA Rudd M Lancsar E Simpson S Teah J. Factors that influence clinicians’ decisions to offer intravenous alteplase in acute ischemic stroke patients with uncertain treatment indication: Results of a discrete choice experiment. Int J Stroke. 2018;13:74–82. doi: 10.1177/1747493017690755 28134031 10.1177/1747493017690755

[26] Meretoja A Weir L Ugalde M Yassi N Yan B Hand P Truesdale M Davis SM Campbell BC. Helsinki model cut stroke thrombolysis delays to 25 minutes in Melbourne in only 4 months. Neurology. 2013;81:1071–1076. doi: 10.1212/WNL.0b013e3182a4a4d2 23946303 10.1212/WNL.0b013e3182a4a4d2

[27] Wu TY Coleman E Wright SL Mason DF Reimers J Duncan R Griffiths M Hurrell M Dixon D Weaver J. Helsinki stroke model is transferrable with “real-world” resources and reduced stroke thrombolysis delay to 34 min in Christchurch. Front Neurol. 2018;9:290. doi: 10.3389/fneur.2018.00290 29760676 10.3389/fneur.2018.00290 PMC5937050

[28] Chollet F. Deep Learning with Python. Manning; 2018.

[29] Guo C Berkhahn F. Entity embeddings of categorical variables. arXiv. 2016. doi: 10.48550/arXiv.1604.06737

[30] Crilly N Blackwell AF Clarkson PJ. Graphic elicitation: using research diagrams as interview stimuli. Qual Res. 2006;6:341–366. doi: 10.1177/1468794106065007

[31] Eakin JM Gladstone B. “Value-adding” analysis: doing more with qualitative data. Int J Qual Methods. 2020:19:1–13. doi: 10.1177/1609406920949333

[32] Archibald MM Ambagtsheer RC Casey MG Lawless M. Using zoom videoconferencing for qualitative data collection: perceptions and experiences of researchers and participants. Int. J. Qual. Methods. 2020;18:1–8. doi: 10.1177/1609406919874596

[33] Salmon J. Qualitative Online Interviews: Strategies, Design, and Skills. Sage; 2014.

[34] Ritchie J Spencer L. Qualitative data analysis for applied policy research. Bryman Burgess, eds. In: Analyzing Qualitative Data. Vol. 11. Routledge; 1994.

[35] Miles M Huberman A Saldana J. Qualitative Data Analysis: A Sourcebook. Sage; 2014.

[36] Mays N Pope C. Rigour and qualitative research. BMJ. 1995;311:109–112. doi: 10.1136/bmj.311.6997.109 7613363 10.1136/bmj.311.6997.109 PMC2550154

