# Supplementary material for: Use of Clinical Pathway Simulation and Machine Learning to Identify Key Levers for Maximizing the Benefit of Intravenous Thrombolysis in Acute Stroke
Source: Stroke. 2022 Jul 15;53(9):2758–67. doi: 10.1161/STROKEAHA.121.038454 (PMC9389935; doi:10.1161/STROKEAHA.121.038454)
Supplement: Supplementary file 2 [file str-53-2758-s002.pdf]

SUPPLEMENTAL MATERIAL

Use of simulation and machine learning to identify key levers for maximising the benefit of thrombolysis in acute stroke

Contents

1 Data 1
2 SSNAP data fields 2
3 General machine learning methodology 4
3.1 Key descriptive statistics of patients used in machine learning model 4
3.2 General machine learning methodology 5
3.2.1 Handling hospital ID 5
3.2.2 Imputation 5
3.2.3 Stratified k-fold validation 6
3.3 Logistic regression 7
3.4 Random forest 7
3.5 Neural networks 9
3.6 Modular network with 1D hospital ID embedding 10
4 Thrombolysis pathway simulation 12
5 Qualitative research 16
5.1 Objectives 16
5.2 Data collection 17
5.3 Data analysis 17
5.4 Results 18
5.4.1 Current attitudes to thrombolysis use 18
5.4.2 Perspectives on simulation and machine learning 20
5.4.3 Potential routes for the implementation of machine learning feedback 22
5.4.4 Anticipated consequences of stroke pathway feedback 23

1 Data

Data was obtained from the Sentinel Stroke National Audit Programme (SSNAP, https://www.strokeaudit.org/), managed through the Healthcare Quality Improvement Partnership (https://www.hqip.org.uk/). SSNAP has near-complete coverage of all acute stroke admissions in the UK (outside Scotland). All hospitals admitting acute stroke participate in the audit, and year-on-year comparison with Hospital Episode Statistics confirms estimated case ascertainment of 95% of coded cases of acute stroke. The NHS Health Research Authority decision tool (http://www.hra-decisiontools.org.uk/research/)

was used to confirm that ethical approval was not required to access the data. Data access was authorised by the UK Healthcare Quality Improvement Partnership (reference HQIP303).

Data was retrieved for all 246,676 emergency stroke admissions to acute stroke teams in England and Wales in SSNAP between 2016 and 2018 (three full years, covering a period where high SSMAP completion rates had been achieved).

2 SSNAP data fields

The data fields provided were as below:

Hospital ID

- *StrokeTeam*: Pseudonymised SSNAP 'routinely admitting team' unique identifier. For emergency care it is expected that each hospital has one stroke team (though post-72 hour care may be reported under a different team at that hospital).

Patient – general

- *PatientUID*: Pseudonymised patient unique identifier
- *Pathway*: Total number of team transfers, excluding community teams
- *S1AgeOnArrival*: Age on arrival aggregated to 5 year bands
- *MoreEqual80y*: Whether the patient is >= 80 years old at the moment of the stroke
- *S1Gender*: Gender
- *S1Ethnicity*: Patient Ethnicity. Aggregated to White, Black, Mixed, Asian and Other

Patient – pathway information

- *S1OnsetInHospital*: Whether the patient was already an inpatient at the time of stroke
- *S1OnsetToArrival_min*: Time from symptom onset to arrival at hospital in minutes, where known and if out of hospital stroke
- *S1OnsetDateType*: Whether the date of onset given is precise, best estimate or if the stroke occurred while sleep
- *S1OnsetTimeType*: Whether the time of symptom onset given is precise, best estimate, not known
- *S1ArriveByAmbulance*: Whether the patient arrived by ambulance
- *S1AdmissionHour*: Hour of arrival, aggregated to 3 hour epochs
- *S1AdmissionDay*: Day of week at the moment of admission
- *S1AdmissionQuarter*: Year quarter (Q1: Jan-Mar; Q2:April-Jun; Q3: Jul-Sept; Q4: Oct-Dec)
- *S1AdmissionYear*: Year of admission
- *S2BrainImagingTime_min*: Time from Clock Start to brain scan. In minutes. "Clock Start" is used throughout SSNAP reporting to refer to the date and time of arrival at first hospital for newly arrived patients, or to the date and time of symptom onset if the patient is already in hospital at the time of their stroke.
- *S2ThrombolysisTime_min*: Time from Clock Start to thrombolysis. In minutes. "Clock Start" is used throughout SSNAP reporting to refer to the date and time of arrival at first hospital for newly arrived patients, or to the date and time of symptom onset if patient already in hospital at the time of their stroke.

Patient – comorbidities

- *CongestiveHeartFailure*: Pre-Stroke Congestive Heart Failure
- *Hypertension*: Pre-Stroke Systemic Hypertension

- *AtrialFibrillation*: Pre-Stroke Atrial Fibrillation (persistent, permanent, or paroxysmal)
- Diabetes: Comorbidities: Pre-Stroke Diabetes Mellitus
- *StrokeTIA*: Pre-Stroke history of stroke or Transient Ischaemic Attack (TIA)
- *AFAntiplatelet*: Only available if "Yes" to Atrial Fibrillation comorbidity. Whether the patient was on antiplatelet medication prior to admission
- *AFAnticoagulent*: Prior to 01-Dec-2017: Only available if "Yes" to Atrial Fibrillation comorbidity; From 01-Dec-2017: available even if patient is not in Atrial Fibrillation prior to admission. Whether the patient was on anticoagulant medication prior to admission
- *AFAnticoagulentVitK*: If the patient was receiving anticoagulant medication, was it vitamin K antagonists
- *AFAnticoagulentDOAC*: If the patient was receiving anticoagulant medication, was it direct oral anticoagulants (DOACs)
- *AFAnticoagulentHeparin*: If the patient was receiving anticoagulant medication, was it Heparin

Patient – National Institutes of Health Stroke Scale (NIHSS)

- *S2NihssArrival*: National Institutes of Health Stroke Scale score on arrival at hospital
- *BestGaze*: National Institutes of Health Stroke Scale Item 2 Best Gaze (higher values indicate more severe deficit)
- *BestLanguage*: National Institutes of Health Stroke Scale Item 9 Best Language (higher values indicate more severe deficit)
- *Dysarthria*: National Institutes of Health Stroke Scale Item 10 Dysarthria (higher values indicate more severe deficit)
- *ExtinctionInattention*: National Institutes of Health Stroke Scale Item 11 Extinction and Inattention (higher values indicate more severe deficit)
- *FacialPalsy*: National Institutes of Health Stroke Scale Item 4 Facial Paresis (higher values indicate more severe deficit)
- *LimbAtaxia*: National Institutes of Health Stroke Scale Item 7 Limb Ataxia (higher values indicate more severe deficit)
- *Loc*: National Institutes of Health Stroke Scale Item 1a Level of Consciousness (higher values indicate more severe deficit)
- *LocCommands*: National Institutes of Health Stroke Scale Item 1c Level of Consciousness Commands (higher values indicate more severe deficit)
- *LocQuestions*: National Institutes of Health Stroke Scale Item 1b Level of Consciousness Questions (higher values indicate more severe deficit)
- *MotorArmLeft*: National Institutes of Health Stroke Scale Item 5a Motor Arm - Left (higher values indicate more severe deficit)
- *MotorArmRight*: National Institutes of Health Stroke Scale Item 5b Motor Arm - Right (higher values indicate more severe deficit)
- *MotorLegLeft*: National Institutes of Health Stroke Scale Item 6a Motor Leg - Left (higher values indicate more severe deficit)
- *MotorLegRight*: National Institutes of Health Stroke Scale Item 6b Motor Leg - Right (higher values indicate more severe deficit)
- *Sensory*: National Institutes of Health Stroke Scale Item 8 Sensory (higher values indicate more severe deficit)
- *Visual*: National Institutes of Health Stroke Scale Item 3 Visual Fields (higher values indicate more severe deficit)

Patient – other clinical features

- *S2INR*: Patient's International Normalised ratio (INR) on arrival at hospital (available since 01-Dec-2017)
- *S2INRHigh*: INR was greater than 10 on arrival at hospital (available since 01-Dec-2017)
- *S2INRNK*: INR not checked (available since 01-Dec-2017)
- *S2NewAFDiagnosis*: Whether a new diagnosis of Atrial Fibrillation was made on admission
- *S2RankinBeforeStroke*: Patient's modified Rankin Scale score before this stroke (Higher values indicate more disability)
- *S2StrokeType*: Whether the stroke type was infarction or primary intracerebral haemorrhage
- *S2TIAInLastMonth*: Whether the patient had a Transient Ischaemic Attack during the last month. Item from the SSNAP comprehensive dataset questions (not mandatory)

Patient – thrombolysis given

- *S2Thrombolysis*: Whether the patient was given thrombolysis (clot busting medication)

Patient – Reason stated for not giving thrombolysis

- *Age*: If the answer to thrombolysis given was "no but", the reason was Age
- *Comorbidity*: If the answer to thrombolysis given was "no but", the reason was comorbidity
- *Haemorrhagic*: If the answer to thrombolysis given was "no but", the reason was Haemorrhagic stroke
- *Improving*: If the answer to thrombolysis given was "no but", the reason was Symptoms Improving
- *Medication*: If the answer to thrombolysis given was "no but", the reason was Medication
- *OtherMedical*: If the answer to thrombolysis given was "no but", the reason was Other medical reason
- *Refusal*: If the answer to thrombolysis given was "no but", the reason was Refusal
- *TimeUnknownWakeUp*: If the answer to thrombolysis given was "no but", the reason was Symptom onset time unknown/wake-up stroke
- *TimeWindow*: If the answer to thrombolysis given was "no but", the reason was that thrombolysis could not be given in the permitted time from onset.
- *TooMildSevere*: If the answer to thrombolysis given was "no but", the reason was Stroke too mild or too severe

Machine learning uses all fields apart from '*Patient – thrombolysis given*' and '*Patient – Reason stated for not giving thrombolysis*' which directly inform whether thrombolysis was given or not.

3 General machine learning methodology

For detailed code see: https://samuel-book.github.io/samuel-1

3.1 Key descriptive statistics of patients used in machine learning model

Table S1 shows key descriptive statistics for patients used in machine learning models (patients arriving at hospital within 4 hours of known stroke onset).

Table S1: Key descriptive statistics for patients used in machine learning models (patients arriving at hospital within 4 hours of known stroke onset).

	All (n=88,928)	Thrombolysis (n=26,257)	No Thrombolysis (n=62,671)
**Thrombolysis given**	0.295	1.000	0.000
**Age on arrival**	75	73	76
**Male**	0.518	0.540	0.509
**Ischaemic (vs. haemorrhagic) stroke**	0.850	1.000	0.787
**Onset to arrival (minutes)**	111	97	117
**Onset known precisely (vs. best estimate)**	0.620	0.820	0.537

**Proportion scanned within 4 hours of arrival**	0.948	1.000	0.926
**Arrival to imaging (minutes, for those scanned within 4 hours)**	117	23	157
**Rankin before stroke**	1.060	0.728	1.199
**NIHSS on arrival**	9.3	11.6	8.4

3.2 General machine learning methodology

Machine learning models were used to predict whether a patient will receive thrombolysis or not, based on a range of patient-related features in SSNAP including which hospital the patient attended. Machine learning models were restricted to those patients arriving at hospital within 4 hours of known stroke onset. And to those 132 units that over three years had at least 300 admissions (average 100 per year) and gave thrombolysis to at least 10 people (in total over the three years).

As we restrict machine learning to those patients who arrive within 4 hours of known stroke onset, the mean use of thrombolysis is 29.5% (*c.f.* 11.8% of all arrivals receiving thrombolysis).

This is a *supervised learning* problem where we train a model using a training set of data that has all the *features* (variables) for the patient and has the *label* of whether a patient received thrombolysis or not. The model is then tested on data that has not been used in training (see section on *stratified k-fold validation* below). We test three different types of machine learning: 1) logistic regression, 2) random forest, and 3) neural networks (with alternative architectures).

3.2.1 Handling hospital ID

We use three alternative ways of handling hospital ID in our models:

1. *One-hot encoding*: A single model is built which predicts use of thrombolysis in all hospitals. Hospital ID is encoded in a vector whose length is equal to the number of hospitals. All values are set to zero, except one value is set to one (one-hot) using the hospital ID as the index. For example if there were 5 hospitals, the one-hot encoding of hospital 2 would be [0, 1, 0, 0, 0]. This one-hot vector is then joined to the rest of the input data. In our models the *one-hot* vector has 132 values, one of which has the value *1* with all others having the value *0.*
2. *Hospital-specific models*: A model is built for each hospital. No encoding of hospital is needed.
3. *Embedding layer* (neural networks only): The hospital ID is first one-hot encoded. The one-hot vector is used as the input into an embedding layer which reduces the one-hot vector to a reduced dimensions size (in this project we reduce the one-hot vector to either a one-dimension or two-dimensional vector). The embedding value is optimised during neural network training so that similar hospitals (from the perspective of decision-making) have embedding values that are similar.

3.2.2 Imputation

Imputation is only used for machine learning (where those patients with unknown stroke onset time, or who have arrived too late for thrombolysis have been removed prior to imputation). We impute either by using an extreme value for numerical fields (e.g. if no time from arrival to scan is recorded we impute the value 9999 mins, or if NIHSS values are missing we impute with the value 0), or if it is a missing categorical value we add a coding category of ‘missing’ for that variable.

The percentage of missing data and the imputation method is shown in Table S2:.

Table S2: Features with missing data, with proportion of rows missing data and imputation method used ('missing': one-hot encode as missing, '0': replace with zero,'9999': replace with 9999).

Feature	% Missing	Imputation method
AF Antiplatelet	79.3	missing
AF Anticoagulent	50.6	missing
AF Anticoagulent Vitamin K	62.5	missing
AF Anticoagulent DOAC	62.5	missing
AF Anticoagulent Heparin	62.5	missing
New AF Diagnosis	72.0	missing
Loc Questions	2.2	0
Loc Commands	2.1	0
Best Gaze	2.7	0
Visual	3.6	0
Facial Palsy	2.1	0
Motor Arm left	2.1	0
Motor Arm right	2.1	0
Motor Leg left	2.2	0
Motor Leg right	2.2	0
Limb Ataxia	4.0	0
Sensory	3.5	0
Best Language	2.3	0
Dysarthria	2.8	0
Extinction Inattention	2.8	0
NIHSS Arrival	5.8	0
Brain Imaging Time (minutes)	0.2	9999
Stroke Type	0.2	missing
TIA in last month	91.0	missing

3.2.3 Stratified k-fold validation

When assessing accuracy of the machine learning models, stratified k-fold splits were used. We used 5-fold splits where each data point is in one, and only one, of five test sets (the same point is in the training set for the four other splits). This is represented schematically in Figure S1. Data is stratified such that the test set is representative of hospital mix in the whole data population, and within the hospital level data the use of thrombolysis is representative of the whole data for that hospital.

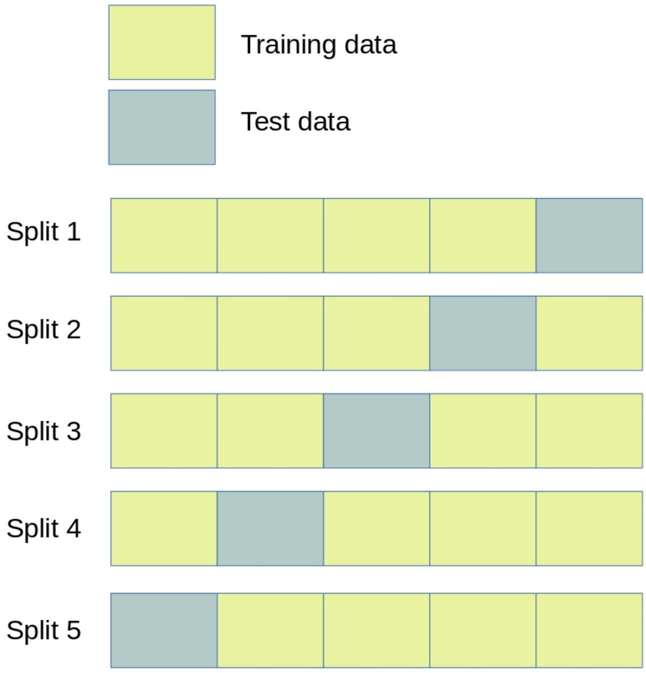

Figure S1: Schematic representation of k-fold splits with five splits.

3.3 Logistic regression

Logistic Regression is a probabilistic model, meaning it assigns a class probability to each data point. Probabilities are calculated using a logistic function:

$$f(x) = \frac{1}{1 + e^{-x}}$$

Here, *x* is a linear combination of the variables of each data point, i.e. $a + bx_1 + cx_2 + ..,$ where *x1* is the value of one variable, x_2 the value of another, etc. The function *f* maps *x* to a value between 0 and 1, which may be viewed as a class probability. If the class probability is greater than the decision threshold, the data point is classified as belonging to class 1 (receives thrombolysis). For probabilities less than the threshold, it is placed in class 0 (does not receive thrombolysis).

During training, the logistic regression uses the examples in the training data to find the values of the coefficients in *x (a ,b ,c, …)* that lead to the highest possible accuracy in the training data. The values of these parameters determine the importance of each variable for the classification, and therefore the decision-making process. A variable with a larger coefficient (positive or negative) is more important when predicting whether or not a patient will receive thrombolysis.

The logistic regression classifier used was from Scikit Learn. Default settings were used.

3.4 Random forest

A random forest is an example of an ensemble algorithm: the outcome (whether or not a patient receives thrombolysis) is decided by a majority vote of other algorithms. In the case of a random forest, these 'other algorithms' are decision trees (each of which is trained on a random subset of examples, and a random subset of features). A random forest is an ensemble of decision trees. Each tree is considered a weak learner, but the collection of trees together form a robust classifier that is less prone to over-fitting than a single full decision tree.

We can think of a decision tree as similar to a flow chart. In Figure S2 we can see that a decision tree is comprised of a set of nodes and branches. The node at the top of the tree is called the root node and the

nodes at the bottom are leaf nodes. Every node in the tree, except for the leaf nodes, splits into two branches leading to two nodes that are further down the tree. A path through the decision tree always starts at the root node. Each step in the path involves moving along a branch to a node lower down the tree. The path ends at a leaf node where there are no further branches to move along. Leaf nodes will each have a particular classification (e.g. receives thrombolysis, or does not receive thrombolysis).

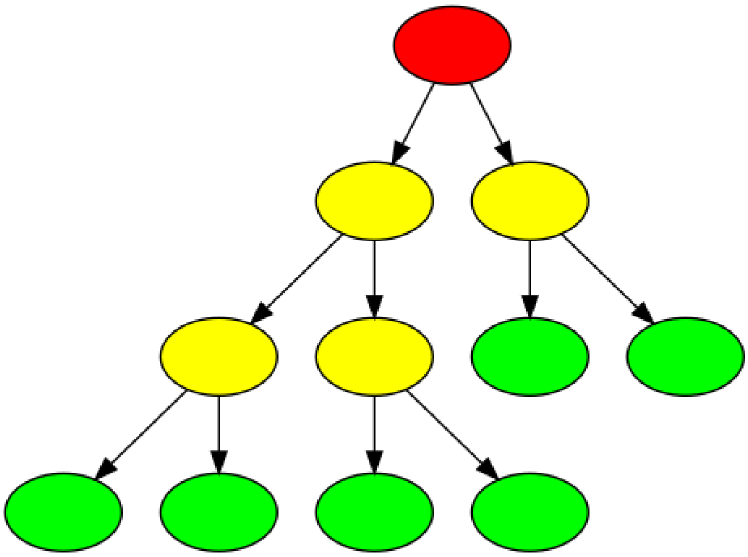

Figure S2: Schematic of a decision tree showing root node (red), splitting nodes (yellow), and terminal leaf nodes (green). A random forest takes the majority vote from a multitude of decision trees.

The path taken through a tree is determined by the rules associated with each node. The decision tree learns these rules during the training process. The goal of the training process is to find rules for each node such that a leaf node contains samples from one class only: the leaf node a patient ends up in determines the predicted outcome of the decision tree.

Specifically, given some training data (variables and outcomes), the decision tree algorithm will find the variable that is most discriminative (provides the best separation of data based on the outcome). This variable will be used for the root node. The rule for the root node consists of this variable and a threshold value. For any data point, if the value of the variable is less than or equal to the threshold value at the root node, the data point will take the left branch and if it is greater than the threshold value it will take the right branch. The process of finding the most discriminative feature and a threshold value is repeated to determine the rules of the internal nodes lower down the tree. Once all data points in a node have the same outcome, that node is a leaf node, representing the end of a path through a tree. Once all paths through the tree end in a leaf node the training process is complete.

A random forest is an ensemble of decision trees. During training the algorithm will select, with replacement, a random sample of the training data, and using a subset of the features will train a decision tree. This process is repeated many times, the exact number being a parameter of the algorithm corresponding to the number of decision trees in the random forest.

The resulting random forest is a classifier that can be used to determine whether a data point belongs to class 0 (does not receive thrombolysis) or class 1 (receives thrombolysis). The path of the data point through every decision tree ends in a leaf node. If there are 100 decision trees in the random forest, and the data point's path ends in a leaf node with class 0 in 30 of the decision trees and a leaf node of class 1 in 70, the random forest takes the majority outcome and classifies the data point as belonging to class 1 (given thrombolysis) with a probability of 0.7 (70/100: number of trees voting class 1 / total number of trees).

The random forest classifier used was from SciKit Learn. Default settings were used, apart from balanced class weighting, where weights for samples are inversely proportional to the frequency of the class label.

3.5 Neural networks

The basic building block of neural networks is the perceptron (Figure S3). Each feature (including a constant/bias feature which usually has the value of one) has an associated weight. The product of each feature multiplied by its weight is summed. The sum is then passed to an activation function. The activation function may leave the input unchanged (often used for a regression output), may use a step function (whereby if the sum of weighted features is less than 0 the output is zero, and if the sum of weighted features is equal to or more than 0 the output is one); may use a logistic function (converting the input into a number between zero or one); or may use an other function. The weights are optimised during the learning process in order to minimise the inaccuracy (loss) of the model. Commonly, optimising is performed according to a variant of stochastic gradient descent, where an example is chosen at random (stochastic), the inaccuracy (loss) is calculated, and the weights are moved a little in the direction which reduced the loss (gradient descent). This learning process is repeated until the model converges on minimum loss.

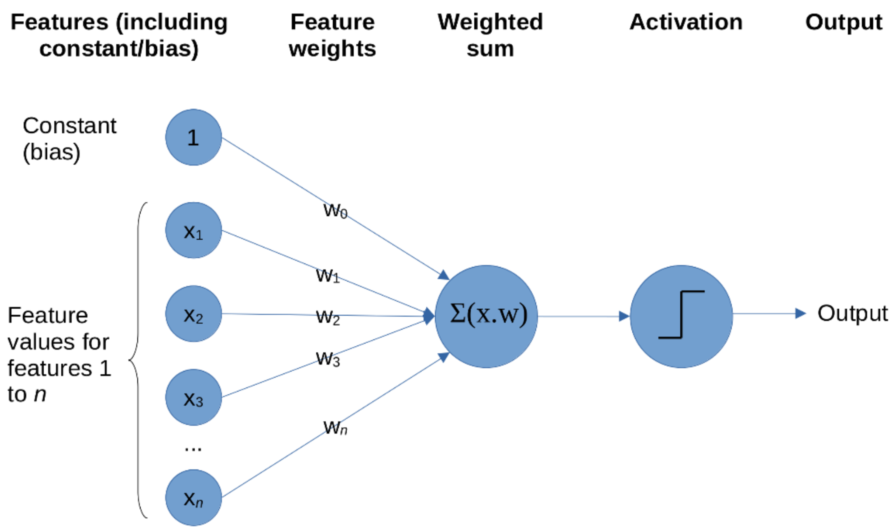

Figure S3: Schematic of a perceptron. Each feature (including a constant) is multiplied by an individual weight for that feature. These feature.weights are summed, and the output passed to an activation function (a simple activation function is a step function whereby if the sum of weighted features is less than 0 the output is zero, and if the sum of weighted features is equal to or more than 0 the output is one).

A neural network is composed of a network of perceptrons, and is sometimes called a *multi-layer-perceptron*. Input features are connected to multiple perceptrons (or neurones) each of which performs a weighted sum of *feature.weights* and passes the output through an activation function. The most common activation function used within a neural network is the *rectified linear unit* (ReLU). Using ReLU, if the weighted sum of inputs is less than zero, the output is zero, and if the weighted sum of inputs is greater than zero then the output is equal to the weighted sum of inputs. This simple function is computationally efficient and is enough for the neural network to mimic non-linear functions. The outputs from a layer in the network are passed as inputs to the next layer. The layers may be of any number of neurones, and may vary between layers (though it is common now to have the same number of neurones in all layers apart from the final layer). The final layer has an activation function depending on the purpose of the network. For example, a regressor network will often leave the weighted sum in the final layer unchanged. A binomial classification network will commonly use logistic/sigmoid activation in the final layer (usually with a single neurone in the output layer), and a multi-class network will often use *softmax* activation where there are as many output neurones as there are classes, and each will have an output equivalent to a probability of 0-1. A standard

approach is to have a 'fully connected' neural network, where each perceptron in the current layer is connected to all perceptrons in the following layer. It is possible to define a custom design with only specific connections chosen.

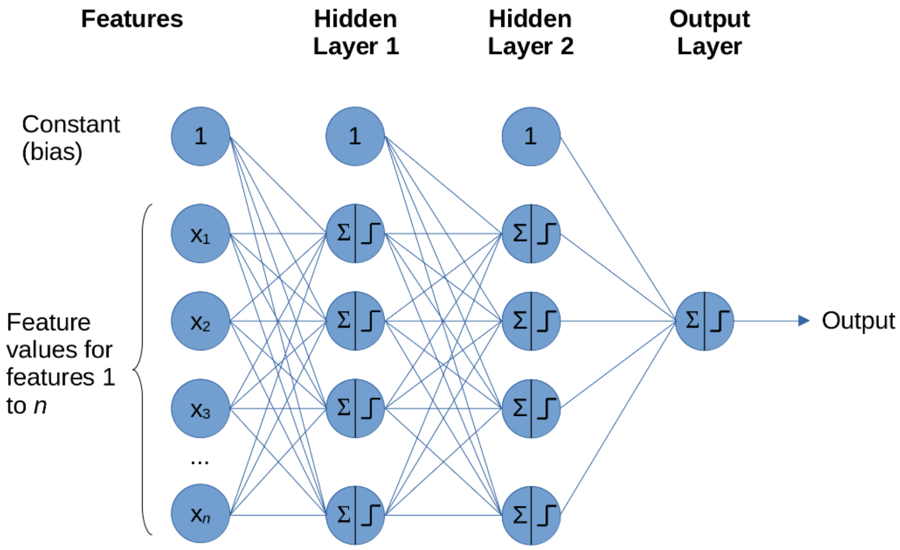

Figure S4: An example neural network. In this 'fully connected' neural network there are as many perceptrons in each layer as there are features (in practice this number may be changed). Each feature is connected to all perceptrons in the first hidden layer, each with its own associated weight.

Figure S4 shows a *fully connected* neural network where all neurones in a layer are connected to all neurones in the next layer. Each neurone sums all inputs multiplied by the weight for each input. This is then commonly passed through an activation function. We use ReLU activation for the hidden layers, whereby all outputs of less than zero are set to zero and all output of greater than zero are unchanged. The final layer is a sigmoid activation layer with an output of 0-1. If the network is well calibrated this output will be the probability of classification of a patient receiving thrombolysis. In order to prevent overfitting of the network we use *dropout* (where 50% of the neurones are randomly excluded in each training run), and *early stopping* (where training of the network is stopped when accuracy of an evaluation data set is no longer improving, and the network weights are rolled back to when the accuracy of the evaluation set was at its highest).

Training a neural network is similar to a perceptron, using methods based on stochastic gradient descent. The additional component in neural network is back-propagation of loss, which distributes loss through the network according to how much individual neurones contribute to the overall loss.

We used the *Keras* neural network library for Python/TensorFlow. General methodology for training and optimising neural networks using Keras is described by Chollet [28].

3.6 Modular network with 1D hospital ID embedding

Embedding uses a subnet (a distinct part of a larger neural network) to convert a categorical variable into a projection onto n-dimensional space [29], and has been shown to be an effective way to train neural networks when using categorical data, while also allowing a measure of similarity/distance between different values of the categorical data. Here we use subnet embedding for three groups of the data available for each patient: 1) hospital ID, 2) patient/clinical characteristics (such as age, gender, stroke symptoms, etc.(see Table S3), and 3) pathway times/timings (such as time from onset to arrival, time to scan, etc). These are either based on a single categorical value (hospital ID), or a group of related data (clinical features, or pathway information). When we convert a set of related data to a smaller dimension space, it may also be known as encoding the data.

Table S3: List of features used for patient clinical embedding.

S1AgeOnArrival	S1Ethnicity_Asian	AFAnticoagulent_missing
S2RankinBeforeStroke	S1Ethnicity_Black	AFAnticoagulentVitK_No
Loc	S1Ethnicity_Mixed	AFAnticoagulentVitK_Yes
LocQuestions	S1Ethnicity_Other	AFAnticoagulentVitK_missing
LocCommands	S1Ethnicity_White	AFAnticoagulentDOAC_No
BestGaze	CongestiveHeartFailure_No	AFAnticoagulentDOAC_Yes
Visual	CongestiveHeartFailure_Yes	AFAnticoagulentDOAC_missing
FacialPalsy	Hypertension_No	AFAnticoagulentHeparin_No
MotorArmLeft	Hypertension_Yes	AFAnticoagulentHeparin_Yes
MotorArmRight	AtrialFibrillation_No	AFAnticoagulentHeparin_missing
MotorLegLeft	AtrialFibrillation_Yes	S2NewAFDiagnosis_No
MotorLegRight	Diabetes_No	S2NewAFDiagnosis_Yes
LimbAtaxia	Diabetes_Yes	S2NewAFDiagnosis_missing
Sensory	StrokeTIA_No	S2StrokeType_Infarction
BestLanguage	StrokeTIA_Yes	S2StrokeType_Primary Intracerebral Haemorrhage
Dysarthria	AFAntiplatelet_No	S2StrokeType_missing
ExtinctionInattention	AFAntiplatelet_No but	S2TIAInLastMonth_No
S2NihssArrival	AFAntiplatelet_Yes	S2TIAInLastMonth_No but
MoreEqual80y_No	AFAntiplatelet_missing	S2TIAInLastMonth_Yes
MoreEqual80y_Yes	AFAnticoagulent_No	S2TIAInLastMonth_missing
S1Gender_Female	AFAnticoagulent_No but	
S1Gender_Male	AFAnticoagulent_Yes	

Hospitals that make similar decisions should end up close to each other in the embedded vector space. As with hospitals, patients that are similar (from the perspective of thrombolysis decision-making) should end up close to each other in the embedded vector space.

Our modular neural networks split data into three subgroups: 1) hospital id, 2) patient/clinical characteristics, 3) pathway times/timings. Each subgroup of data is processed by a neural subnet to produce a vector. The architecture may be set to produce a vector of any number of dimensions - here we use one and two values per subnet output vector. The output from the subnets is combined in an additional layer in the neural network, the concatenation layer which outputs a sigmoid probability of receiving thrombolysis.

When the subnets output a single value, this will condense each of the subgroups down to a single value that will be used in the final layer to determine probability of thrombolysis. This allows, for example, ranking of patients suitability for thrombolysis determined by a consensus view from all hospitals, and similarly allows ranking of hospitals by propensity to give thrombolysis, independent of their own patient population. When two or more output values are used for each subnet, this allows for more complex interactions between patients and hospitals to be represented, and offers the potential to cluster similar hospitals or patients by location of their output vectors.

Figure S5 shows a schematic representation of the modular neural network with 1-dimensional embedding (hospital, pathway, and clinical features each get converted to a 1-dimensional vector - a single value), before being combined in a final concatenation layer with sigmoid activation. Pathway data and clinical data each have one hidden layer before being embedded by sigmoid activation.

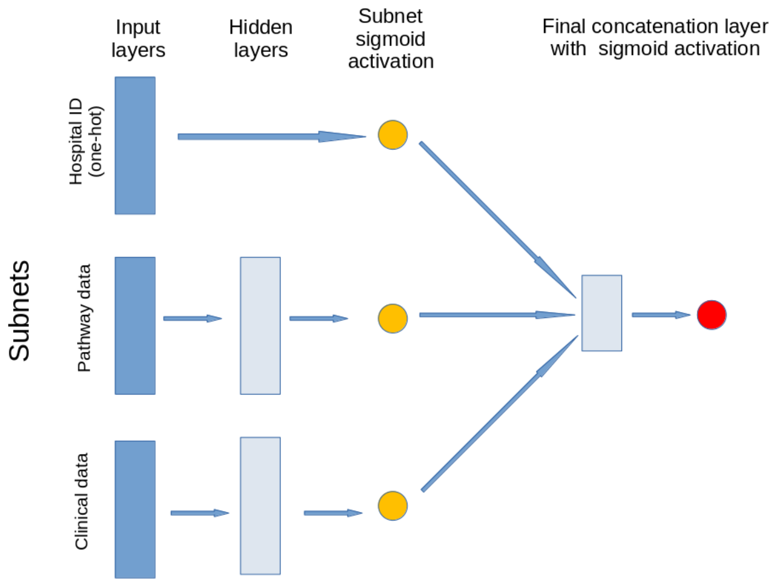

Figure S5: Schematic representation of the modular neural network with 1D embedding.

4 Thrombolysis pathway simulation

The pathway simulation is constructed in Python, using NumPy [16]. Code for the model may be found in the accompanying Jupyter Book in section: https://samuel-book.github.io/samuel-1/pathway_sim/pathway_code.htmlThe stroke pathway simulation models the passage of a cohort of patients through a hospital's stroke pathway. Timings in the simulation are sampled from distributions using NumPy's `random` library which uses the PCG-64 pseudo-random number generator. These distributions may be based on observed timings or may be 'what if?' scenarios such as 'what if arrival-to-scan time was consistently 15 minutes?' All process times are sampled from log normal distributions (see below for fitting of distributions).

Figure S6 shows the distribution of process times across all hospitals. All timings show a right skew.

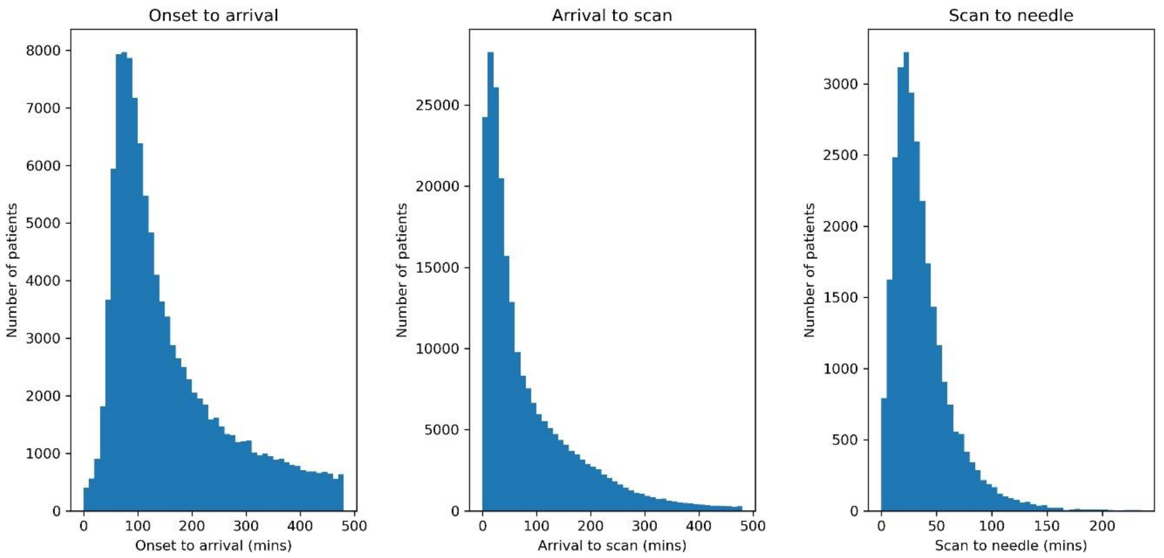

Figure S6: Histograms of distribution of timings for onset-to-arrival (left), arrival-to-scan (middle) and scan-to-needle (right).

Ten candidate distribution types were fitted to the data. All three process times were best fitted by a log-normal distribution (chosen by lowest chi-squared; distributions were fitted to 10K bootstrapped samples for each process time). Figure S7 to Figure S9 show log-normal distribution fits for the three process times.

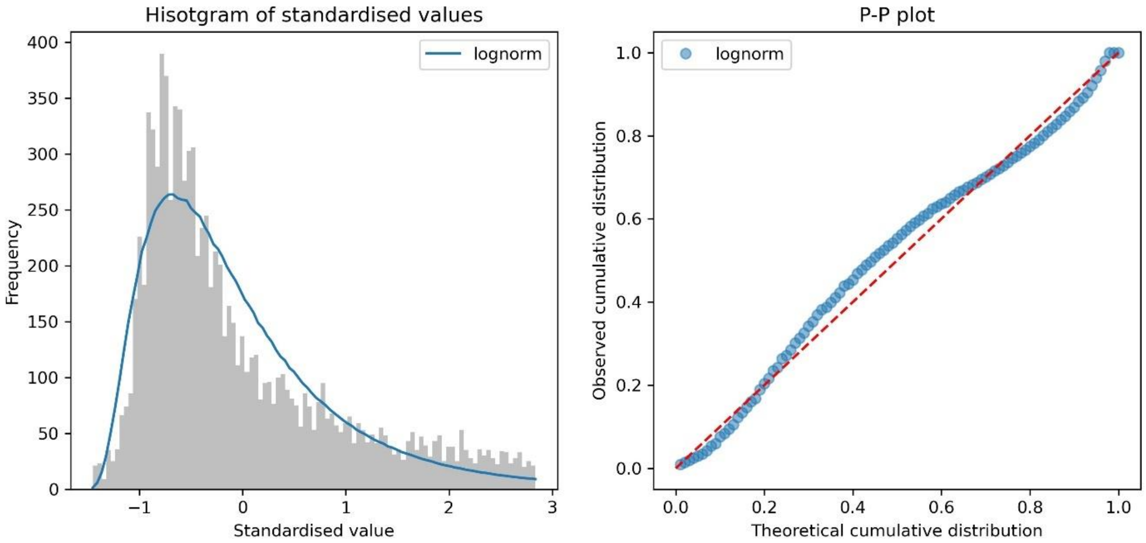

Figure S7: Distribution fitting to onset-to-arrival times. Left: Histogram of standardised values and fitted distribution (lognormal). Right: P-P plot of actual vs. theoretical (fitted) probabilities.

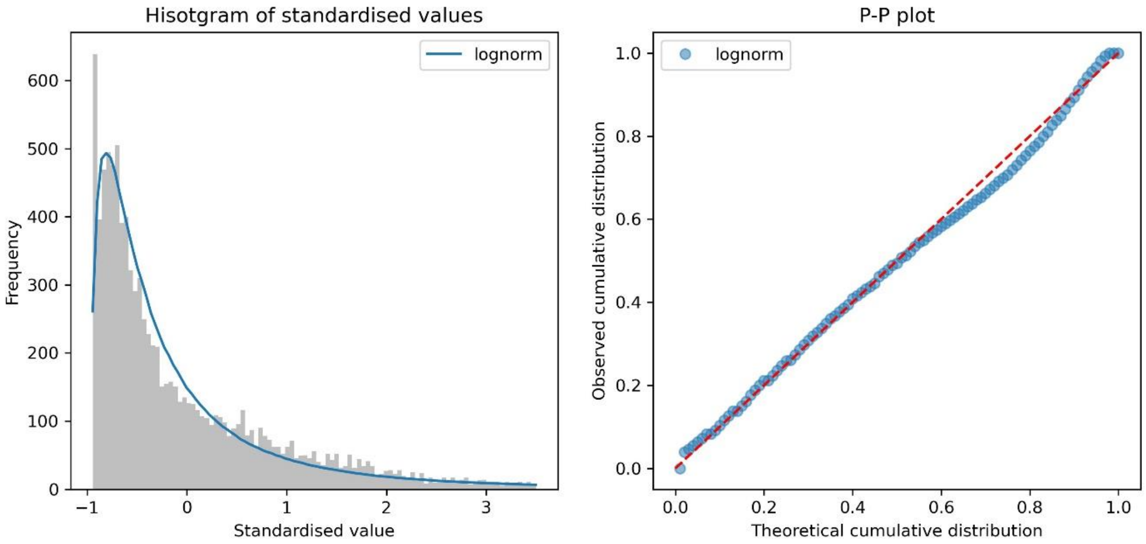

Figure S8: Distribution fitting to arrival-to-scan times. Left: Histogram of standardised values and fitted distribution (lognormal). Right: P-P plot of actual vs. theoretical (fitted) probabilities.

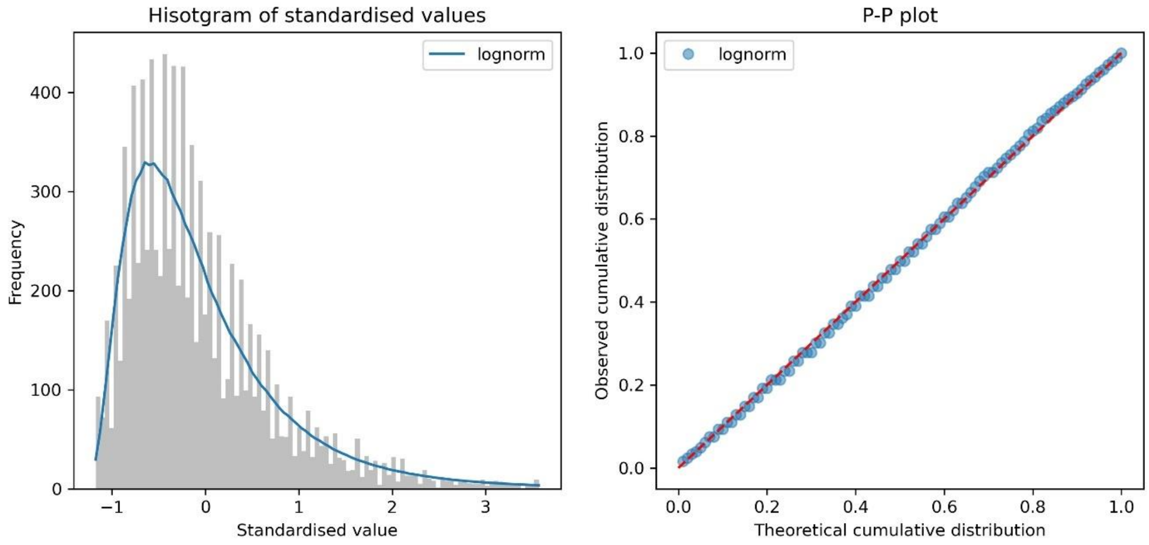

Figure S9: Distribution fitting to scan-to-needle times. Left: Histogram of standardised values and fitted distribution (lognormal). Right: P-P plot of actual vs. theoretical (fitted) probabilities.

The key process steps in the pathway are shown in Figure S10. Patients can leave the pathway at each step if their pathway durations exceed the permitted time limits, or they become ineligible for treatment. Only patients that satisfy all restrictions continue along the full length of the pathway and receive thrombolysis. The outcome is then calculated as a probability of having a good outcome (mRS 0-1). If the patient does not receive thrombolysis the probability of a good outcome is the baseline probability of a good outcome in the population age group (aged under 80 years, or aged 80+ years). If the patient received thrombolysis then the probability of a good outcome is based on age group and time to treatment.

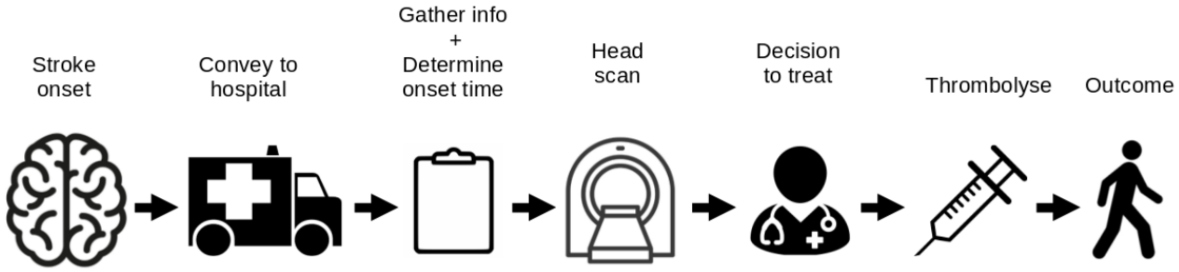

Figure S10: Schematic representation of the stroke pathway as simplified for the simulation. Patients can leave the pathway at each step if their pathway durations exceed the permitted time limits, or they become ineligible for treatment. Only patients that satisfy all restrictions continue along the full length of the pathway.

The probability of having a good outcome (mRS0-1) is modelled based on time to treatment according to a meta-analysis of clinical trials [1], as shown in Figure S11.

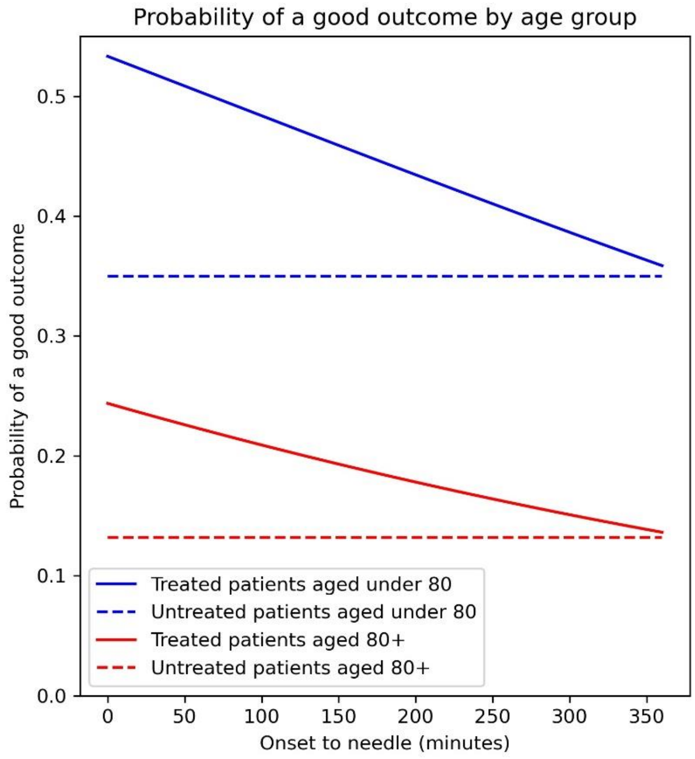

Figure S11: Effect of time to treatment (onset-to-needle time) on the clinical benefit (probability of an outcome with modified Rankin Scale score of 0-1) from thrombolysis, as derived by a meta-analysis of clinical trials (Emberson et al. 2014).

Individual patient pathways are modelled within a NumPy array for each hospital. The following fields are populated based on sampling from distributions:

- Patient aged 80+ (Boolean, based on Bernoulli distribution)
- Allowable onset-to-needle time (minutes, specified: may depend on age)
- Stroke onset time known (Boolean, based on Bernoulli distribution)
- Onset-to-arrival time is less than 4 hours (Boolean, based on Bernoulli distribution)
- Stroke onset time known and onset-to-arrival time is less than 4 hours (Boolean, calculated based on values above)
- Onset-to-arrival time (minutes, based on log normal distribution)
- Arrival-to-scan time is less than 4 hours (Boolean, based on Bernoulli distribution)
- Arrival-to-scan time (minutes, based on log normal distribution)
- Time left to thrombolyse (minutes, calculated based on values above)
- Stroke onset time known and time left to thrombolyse (Boolean, calculated based on values above)
- Ischaemic stroke (if they are filtered at this stage. Boolean, based on Bernoulli distribution)
- Assign eligible for thrombolysis (Boolean, based on Bernoulli distribution. Will receive thrombolysis if scanned within permitted treatment time)
- Thrombolysis planned (Boolean, calculated: scanned within time and eligible)
- Scan-to-needle time (minutes, based on log normal distribution)

Each hospital has a base scenario based on its current performance. A statistical summary of these parameters across 132 hospitals is shown in Table S4 shows a description of the ranges used for model parameters. Full parameters for each hospital may be found at: https://samuel-book.github.io/samuel-1/pathway_sim/base_parameters.html

Table S4: A summary of ranges of parameters determined and used in the pathway simulation model (each of 132 hospitals has it's own values that are used to define the distributions that individual patients will be sampled from).

Parameter	count	mean	std	min	25%	50%	75%	max
thrombolysis_rate	132	0.115	0.035	0.015	0.093	0.110	0.133	0.243
admissions	132	573	277	101	378	544	755	2039
80_plus	132	0.426	0.056	0.292	0.387	0.428	0.458	0.576
onset_known	132	0.666	0.134	0.345	0.574	0.642	0.753	0.988
known_arrival_within_4hrs	132	0.601	0.090	0.257	0.558	0.609	0.662	0.814
onset_arrival_mins_mu	132	4.572	0.098	3.768	4.530	4.580	4.629	4.763
onset_arrival_mins_sigma	132	0.560	0.121	0.435	0.517	0.547	0.586	1.801
scan_within_4_hrs	132	0.948	0.030	0.847	0.933	0.954	0.969	1.000
arrival_scan_arrival_mins_mu	132	3.280	0.409	1.666	3.030	3.310	3.572	4.236
arrival_scan_arrival_mins_sigma	132	0.941	0.221	0.549	0.788	0.891	1.034	1.751
onset_scan_4_hrs	132	0.875	0.043	0.627	0.853	0.877	0.904	0.962
eligable	132	0.338	0.083	0.111	0.276	0.332	0.399	0.532
scan_needle_mins_mu	132	3.426	0.342	2.573	3.204	3.426	3.677	4.409
scan_needle_mins_sigma	132	0.690	0.156	0.366	0.579	0.662	0.793	1.283

Using these populated fields, the following process is carried out to calculate the probability of a good outcome.

1. Clip onset to thrombolysis time to maximum allowable onset-to-needle time (minutes)
2. Set baseline probability of good outcome based on age group (scalar probability)
3. Convert baseline probability good outcome to odds (scalar odds)
4. Calculate odds ratio of good outcome based on onset-to-needle time (scalar odds ratio)
5. Patient odds of good outcome if given thrombolysis (scalar adjusted odds)
6. Patient probability of good outcome if given thrombolysis (scalar adjusted probability)
7. Clip patient probability of good outcome to minimum of zero (scalar adjusted probability)
8. Individual patient good outcome if given thrombolysis (Boolean)*
9. Individual patient good outcome if not given thrombolysis (Boolean)*

*Net population outcome is calculated here by summing probabilities of good outcome for all patients, rather than using individual outcomes. These columns are added for potential future use.

Replication was set to 100 x one year runs. This gave a precision (95% confidence limit) of less than 5% of mean values for thrombolysis use and predicted number of additional good outcomes.

5 Qualitative research

5.1 Objectives

The overall objective of the qualitative research was to understand influence of modelling, including the use of machine learning techniques, in the context of the national audit, in order to support efforts to maximise the appropriate use of thrombolysis and reduce unnecessary variation.

Specifically the aims were to:

1. Explore current understanding and rationale for the use of thrombolysis for ischaemic stroke, in order to establish reasons for the variance in the use and speed of thrombolysis.

2. Understand physician perspectives on simulation and machine learning feedback, to influence how simulation can be incorporated into the Sentinel Stroke National Audi Programme (SSNAP) to have a positive impact on practice.
3. Identify potential routes for the implementation of machine learning feedback, to inform and improve future stroke management.
4. Explore how physicians interpret the potential consequences of following changes in pathway suggested by simulation.

5.2 Data collection

To pilot our interview approach, we undertook a face-to-face group interview with a small group of medical registrars on a regional rotation that included stroke, in their clinical setting. Having given written consent to interview, a senior modeller provided the registrars with a demonstration of the modelling process and outcomes, prior to the qualitative researchers piloting the topic guide. Feedback from the stroke physicians suggested that this approach was appropriate and produced data that was fit for purpose.

Our approach was subsequently modified for remote delivery, with all interviews conducted via MS Teams, Skype or Zoom, dependant on the media that was allowed in each NHS Trust. At the beginning of each interview participants watched a 10-minute video made by the senior modeller that contained examples of the process and outcomes of the machine modelling and pathway analysis, as a stimulus for discussion [30]. The topic guide was then used to elicit participants' own experiences of thrombolysis and perspectives on machine learning, alongside observations of group interactions and clinical settings [31].

During the interviews, we collected data about physicians' backgrounds, their attitudes to thrombolysis and their understanding of variance, their perspectives on machine learning, and potential loci for the implementation of machine learning feedback (within and beyond SSNAP), established the physicians' views on possible unintended consequences, which may result from changing the acute stroke pathway, and potential means of mitigation. Our fieldnotes reflected the challenges of conducting interviews via video, with physicians often in clinical settings and sometimes wearing Personal Protective Equipment (PPE), as well as capturing the dynamics between physicians, who were also remote working from each other [32,33].

Towards the end of the project, and during the third lockdown for COVID-19, we undertook an online discussion of our results with a small group of physicians (N=3) who were identified via an annual meeting for trainees, organised by the British Association for Stroke Physicians (BASP). The modellers presented a further set of outputs from their analyses, and the discussion focused on how additional modelling outputs might be used to facilitate quality improvement and inform service delivery.

The number of participants recruited was lower than originally planned. This was due to NHS focus on coping with Covid, and also reflected more challenge in accessing staff from units with lower thrombolysis use. The interview participants were also skewed heavily towards medics. In any future study we recommend having more qualitative resource in order to help recruit more staff, especially from units with lower thrombolysis use, and to broaden the interview base to include more non-medic staff (e.g. more specialist stroke nurses).

5.3 Data analysis

Interview data was transcribed by an independent GDPR-compliant transcriber, and fieldnotes were written up by the two researchers. All data were anonymised and managed in Nvivo for Teams (https://www.qsrinternational.com/). Both researchers read all the transcripts to develop preliminary ideas and understanding. We developed these ideas alongside further re-reading of the transcripts, using a

Framework Analysis aligned with the four broad exploratory objectives of the study, but crucially with an openness to any new insights from the physicians [34, 35]. Analytical summaries across multiple cases were created independently by both researchers, and used to explore the data. We held repeat discussions to develop the analysis, looking for negative cases, and resolving differences of opinion about interpretation [36]. In this way, we were able to examine these physicians' accounts of their use of thrombolysis and orientation to machine learning and simulation. As our analyses developed, we also discussed our findings with members of the wider research team.

5.4 Results

We recruited nineteen participants, who took part in three individual and five group interviews. Eleven of the participants were consultants (specialising in stroke, neurology or elderly care) with four stroke registrars and one specialist stroke nurse. Ten participants were male and nine were female (see Table S5).

Table S5: Qualitative research participants

Thrombosis	Interviews
Low (Site A)	Group: 2 Stoke Consultants (2 x M) + I Specialist Stroke Nurse (1 x F)
Low (Site B)	Single: Consultant Stroke and elderly medicine (M)
Low (Site C)	Group: 3 Stroke Consultants (1M/2F)
Middle (Site D/Pilot)	Group: 4 Registrars (4F) (Pilot interview)
Middle (Site E)	Single Consultant Geriatrician (M)
Middle (Site F)	Single: Stoke Consultant(M)
High (Site G)	Group: 3 Consultants: Stroke & Geriatrician (1M/2F)
High (Site H)	Group: 3 Consultants: Stroke, Neurologist, Geriatrician (2M/1F)

5.4.1 Current attitudes to thrombolysis use

Physicians working in hospitals with lower thrombolysis rates were more likely to suggest that a significant barrier to thrombolysis was the delayed presentation of patients, which could be magnified by suboptimal ambulance services:

> "A lot of patients present outside the window of thrombolysis at the hospital." (Site B)

> "I think we rarely hit the 11% percent national numbers, probably because patients come just outside the thrombolysis window." (Site A)

Those working in hospitals with lower thrombolysis rates were more likely to report that their patients were 'different' to those presenting at other centres – in terms of rurality, ethnicity, frailty, or socio-demographic factors:

> "We've a slightly older population… we've slightly more bleeds than infarcts…we're a slightly larger geographical area, so sometimes people are a bit delayed getting to hospital and we operate across two sites as well." (Site C)

The above physician also highlighted that because of these complexities, decision making about thrombolysis was the most difficult part of their job.

While population differences were also acknowledged by physicians at higher thrombolysing centres, they were more likely to articulate the centrality of patient heterogeneity in their decision making:

> Consultant 1:“I wouldn’t be giving thrombolysis for various reasons… They’re often late, or got a very mild deficit, or they’ve got something that makes you feel extra wary about treating them… we’ve got a population that’s increasingly frail, they’ve got multiple comorbidities… [but] every patient is unique.”
> Consultant 2: “We all have different approaches, I say to myself the first question is, if I don’t thrombolyse this patient, what is the worst neurological outcome they could have? What is the disability going to be? And then the next question is how far are we down the time pathway, what’s the risk of bleeding here? And then, what are the little things that feed into pros and cons, how does that alter the equation from a standard patient? is it that the benefits are going to outweigh the risks, how finely balanced is that decision?”
> Consultant 3: “The days of people being textbook strokes are long gone… we don’t see them… we don’t have a blanket policy. We eyeball them. And if they look dodgy we park them and work out what’s going on, if they don’t look dodgy, we go straight to the scanner.” (Site H)

Those in mid-rate thrombolysis centres suggested that some of the delays in patient presentation could be mitigated through treatment by stroke physicians rather than generalists, or by the involvement of a Specialist Stroke Nurse:

> “We typically have a more deprived population, so accessing health care and time to hospital [and] our ambulance service is not as good…”, “a burden of disease due to deprivation… we do see a lot of young strokes… smoking, drinking, drug abuse… expertise is important there, so if you looked at our patients… the ones that had been thrombolysed under 30 minutes… nearly all of them had been managed by a stroke registrar or a geriatrics registrar or a geriatrics consultant.” (Site E)

> “[a] stroke nurses being there increases the speed…”(Site D)

Those physicians currently working in centres with low or medium thrombolysis rates seemed more likely to emphasise the equipment that they lacked, and which they perceived would improve the accuracy and speed of their decision making.

Similarly, physicians working in hospitals with higher thrombolysis rates suggested that their higher rates were due to access to scans and other specialist facilities, as well as 24 hour stroke services:

> “We’re a big teaching hospital… that’s also got a trauma centre” (Site H)

> "Thrombolysis is done by registrars with consultation on the phone with some access to the imaging for the consultant… there is no dedicated stroke team at night… we have a big variation between out of hours and in hours door to needle time… it's 38 minutes, out of hours it's 89 minutes… don't thrombolyse wake up stroke… MRI… perfusion scan… we don't have the facilities." (Site F)

> "On SSNAP data, we are one of the top performing units in the country and that has happened through years of planning and hard work, where we take direct admissions, twenty-four seven, we don't do remote assessments… it's always face to face assessments by consultant… with a specialist nurse, to see a patient, etc. And we have access to scans directly, including vascular imaging…" (Site G)

Thus, the provision of more diagnostic tools was perceived as enabling a more nuanced approach to risk management - that went beyond tallying risk factors, and individualised patient care for more 'marginal' case:

> "If I might manage a level of uncertainty about the onset time and some other characteristics, medications, for example, a slightly imperfect history that I have, if it's a very severe stroke, it's going to be a disabling stroke and I feel that the risks are outweighed by the benefits... I think that stroke severity and my perception of the ability to benefit from thrombolysis will then weigh into how much uncertainty I'm able to cope with, with the other things."(Site G)

Although the sample interviewed was small, they were diverse in their attitudes to thrombolysis use.

5.4.2 Perspectives on simulation and machine learning

Physicians who identified as confident thrombolysers had an initial scepticism of both the premise and methods employed in the simulations they were shown, although this scepticism was later dispelled:

> "The first thought that came to mind was [with the modelling] an innate assumption that doing more thrombolysis is a good thing... So, your machine learning may tell us how to do a lot of people who possibly don't need it, possibly. I'm not saying that's necessarily what you're going to do, but it's where it might go if we just say 'more is better'." (Site H)

Physicians who both worked alone and were interviewed in isolation were more anxious about how the simulation might be used to hold them to account for their decision-making and identified perceived risks:

> "I'd be suspicious if such a tool was available and a patient wasn't thrombolysed, then that might involve the lawyers and the legal teams."(Site C)

> "I think safety would be the top thing, isn't it, it's got to be a hundred percent safe and I think if you are close to a hundred percent safe, if you can show that, if you can show that

it’s safe and it doesn’t cause any negative outcomes for patients, then -and it also enhances patient care by speeding the process up, then I think you’ve won. If there’s doubts about its safety, even if it does speed things up, people aren’t going to trust it… clinicians are always wary about litigation, as well… some of this software could be used retrospectively… it could lead to decision-making being criticised retrospectively.” (Site E)

Those who had the benefit of working in a team with both a culture of collaboration and professional challenge were more inclined to see machine learning as a resource to draw upon for their own decision-making. For example, with those in low thrombolysing centres suggesting that it might augment their decision-making, while those in higher thrombolysing centres viewing it as a positive challenge to inherent assumptions that they might have developed:

“I think it would be a help if there was a patient where, you know, maybe somebody else would have thrombolysed them and we might see something that we weren’t doing that we would then, you know, implement as an action, if there was something clearly that we could be doing, you know, that would improve the rates.” (Site B)

“if you have a computer model you might get out these things out of, well, at least you think about it if the computer says something, then you have to have a strong kind of argument to refuse, to say no. [laughs] To say, well, the computer says, well the modelling comments that they should be thrombolysed, why do you say it’s not thrombolysed, you can’t just say oh, because he’s old or whatever.” (Site F)

“it would be useful to know what would somebody else do. Now, whether that’s presented as a number or as a likelihood for thrombolysis… the hospitals…” (Site F)

In addition, those in the highest thrombolysing centre also thought that the modelling outputs could extend their quality improvement initiatives:

“It’s just a tool, isn’t it, it’s just another tool. We would never – you’d never base your decision on what the machine said! I mean, not until it’s like, you know, the Star Trek computer!... You’re generating data for improving a process and for understanding of process, so it’s very helpful for that… And it might be useful to beat the managers and say we need help with this, that and the other, but then any audit does that.” (Site H different physician).

Some participants suggested additional variables that they would like to see included in the modelling:

“There are factors there which we would use in our decision-making process which are not listed as inputs…active bleeding, head injuries, blood pressure, whether the patient assents or consents [inputs]are insufficient and superficial…”(Site G)

“The other thing that feeds in is that not everyone’s comfortable looking at CT heads and some people are waiting for that to be reported and I think that can add considerable time…

> especially down here, the radiology registrar is not always based in this hospital. So, they cover the whole of the [area] and they might be based in [other centre], whereas in the daytime you can just walk round the corner and speak to the radiologist reporting the scan, and say "What do you think, is it OK", or call the consultant on call. But I think if you're, for example, a med reg in another speciality thrombolysing at night, you wouldn't have that confidence to say that and then having to call up a radiology reg on call in another hospital all takes time, doesn't it… Say, at worst, half an hour." (Site D).

Centres with middle thrombolysis rates were keen to see the outcomes of employing machine learning included in the outputs:

> "How have you extrapolated your outcome data?... what I think would be useful to know is within that people of decision makers, who is making the decisions?... [I would want to see] median times with clear confidence intervals would be most useful...diagnosis at discharge comparative to decision making at the time." (Site D)

> So I think that is, kind of, disabilities should be part of that pathway, some type of assessment for that, for instance, the things which I do is I have to try and identify a link between the disability they get and patient kind of function - that would be helpful." (Site F)

Perspectives on simulation and machine learning varied by the size and type of unit that the physicians worked in, with some participants welcoming the addition of modelling to their decision making tool kit, with others worried about the loss of their agency.

5.4.3 Potential routes for the implementation of machine learning feedback

Across centres, there was an understanding that modelling had the potential to identify which changes a particular centre could invest in to improve their stroke pathway:

> "Tell us we should do our scans quicker or hurry up with our CTAs"…"Placing them on the scanner table rather than wheeling them round… simple things."(Site H)

> "the SSNAP data we have is great, but it's difficult to apply that to solutions locally. Whereas if you could apply the modelling to a local set-up and find out where the delays are consistently across a number of cases, rather than just looking at one case…if you do that across hundreds of cases in the same centre, then you find local solutions to increase speed." (High Site H, different physician).

When asked to identify the potential routes by which machine learning might inform or improve future stroke management, physicians replied with suggestions that matched particular issues with which they were grappling.

Those in low thrombolysing centres wanted a tool that could help them to improve care with a particular patient or type of patient, via a prototypical patient:

> "[I] Would value a prototype patient… where it showed you which hospital would or wouldn't thrombolyse… I would trust the data… we could get some advice on where to improve… there might be some big gains from that, if we did it." (Site B)

> "If we had the information that over the country [about older frail people from care homes], it would probably give those hospitals that are more cautious, more confidence to give that thrombolysis" (Site D).

> "People are quite afraid of the risk of bleeding and things like that. If they produce a type of individualised risk and benefit for the patient, on the information that's provided on algorithm, that would be very helpful… and also the ability to be updated quickly, that would be very helpful, because the texts change every year and then sometimes you can't keep up with all those protocols and pathways." (Site H)

Physicians working in the highest thrombolysing unit, who expressed greater familiarity with SSNAP as well as other performance indicators, wanted a more sophisticated instrument that could compare treatment across consultants or centres:

> Consultant 1: "internally we tend to look at consultant level data, just by looking at the thrombolysis data and picking that apart. But obviously the numbers are small, so the data can be quite varied… but I don't mind seeing it at consultant level information as well as, then, hospital."
> Consultant 2: "You take a prototypical patient and apply them to the algorithms that you've constructed for our hospital and see what pops out the other end… These things rarely provide an answer; they just point you to something you can reflect on…" (Site H)

Perspectives on the potential routes for the implementation of machine learning feedback were informed by physicians' beliefs about their current needs, with the idea of a prototype patient proving popular. However, there was variance in beliefs about what variables should be included, and whether its objective should be direct patient care or as a quality improvement tool.

5.4.4 Anticipated consequences of stroke pathway feedback

Two physicians, both of whom worked on their own, in lower thrombolysing centres, were sceptical about the consequences of changes to the stroke pathway. Having identified that they found decision making about thrombolysis difficult, both then questioned the evidence base for increasing the rate of thrombolysis:

> "I do think it's about, kind of, the personality of the person deciding it, it is very subjective, is thrombolysis, I mean, I know we have all the guidelines as to who we should and shouldn't thrombolyse, but, you know, some consultants will aggressively continue to reduce the blood pressure with as much IV medication as they can until they can thrombolyse, others will say, well, you know, a few doses and if it doesn't come down, OK, it's probably not meant to be, so yeah. And you know, I think I personally just sort of very much stay within the exact rules for whether you should or shouldn't thrombolyse." (Site B)

> "more thrombolysis doesn't mean better care... when I hear of hospitals that are thrombolysing,… twenty odd percent, I do sometimes question them. Are they really thrombolysing strokes? Is the clinical diagnosis of stroke really robust enough or are they

thrombolysing mimics and then putting that into their SSNAP data anyway, just to make them look good. And then their mortality rates are lower because they've thrombolysed non-strokes anyway. So, some of me is – I'm a bit cynical with, of the SSNAP data sometimes, from some of the sites that appear to be doing really well." (Site E)

In contrast, those in higher thrombolysing centres had a more balanced perspective on the perceived benefits of implementation of machine learning in the stroke pathway but identified the likely enduring challenges of thrombolysis decision making:

> Consultant 1:"There's been a gazillion studies looking at how to give TPA quicker, so the question, I think, for you guys, is what's going to be different about this, compared to everybody else's that tells us to get ready, get the ambulance there quicker, be more streamlined, have a checklist, der, der, der, you know, what's going to be different?"
> Consultant 2: "Outcomes data with a comparator is a disaster, what does it mean?... I think you're going to end up with a league table, but basically we already have one with SNAPP."
> Consultant 1: "The implementation of artificial intelligence and automated reporting of scans would change the picture, would change the landscape, let's say, of the speed of thrombolysis."
> Consultant 2: "To be fair, the only aspect of machine learning I can see in this is the thrombolysis decision making process. The rest is all straightforward factors…. The only two parts machine learning is going to help is if the machine can actually interpret the head scan for us, which is really part of the decision to treat or not treat, and that's the only real machine learning aspect of this, the rest is not… your decision to treat or not treat… That's the difficult part. That's the grey area where everyone does a different thing." (Site H)

Participants were clearly curious about machine learning and they welcomed the opportunity to discuss its potential benefits for stroke pathway feedback. Findings suggest that stroke physicians have doubts and concerns about the ability of machine learning to improve the pathway, suggesting that further dialogue is required.
